# Virtual Care and Emergency Department Use During the COVID-19 Pandemic Among Patients of Family Physicians in Ontario, Canada

**DOI:** 10.1001/jamanetworkopen.2023.9602

**Published:** 2023-04-28

**Authors:** Tara Kiran, Michael E. Green, Rachel Strauss, C. Fangyun Wu, Maryam Daneshvarfard, Alexander Kopp, Lauren Lapointe-Shaw, Lidija Latifovic, Eliot Frymire, Richard H. Glazier

**Affiliations:** 1 Department of Family and Community Medicine, St Michael’s Hospital, University of Toronto, Toronto, Ontario, Canada; 2 MAP Centre for Urban Health Solutions, Li Ka Shing Knowledge Institute, St Michael’s Hospital, Toronto, Ontario, Canada; 3 ICES Central, Toronto, Ontario, Canada; 4 Institute of Health Policy, Management and Evaluation, University of Toronto, Toronto, Ontario, Canada; 5 Department of Family Medicine, Queen's University, Kingston, Ontario, Canada; 6 Health Services and Policy Research Institute, Queen’s University, Kingston, Ontario, Canada; 7 ICES Queen’s, Kingston, Ontario, Canada; 8 Dalla Lana School of Public Health, University of Toronto, Toronto, Ontario, Canada; 9 Department of Medicine, University of Toronto and University Health Network, Toronto, Ontario, Canada

## Abstract

**Importance:**

The COVID-19 pandemic has played a role in increased use of virtual care in primary care. However, few studies have examined the association between virtual primary care visits and other health care use.

**Objective:**

To evaluate the association between the percentage of virtual visits in primary care and the rate of emergency department (ED) visits.

**Design, Setting, and Participants:**

This cross-sectional study used routinely collected administrative data and was conducted in Ontario, Canada. The sample comprised family physicians with at least 1 primary care visit claim between February 1 and October 31, 2021, and permanent Ontario residents who were alive as of March 31, 2021. All residents were assigned to physicians according to enrollment and billing data.

**Exposure:**

Family physicians’ virtual visit rate was the exposure. Physicians were stratified by the percentage of total visits that they delivered virtually (via telephone or video) during the study period (0% [100% in person], >0%-20%, >20%-40%, >40%-60%, >60%-80%, >80% to <100%, or 100%).

**Main Outcomes and Measures:**

Population-level ED visit rate was calculated for each stratum of virtual care use. Multivariable regression models were used to understand the relative rate of patient ED use after adjusting for rurality of practice, patient characteristics, and 2019 ED visit rates.

**Results:**

Data were analyzed for a total of 13 820 family physicians (7114 males [51.5%]; mean [SD] age, 50 [13.1] years) with 12 951 063 patients (6 714 150 females [51.8%]; mean [SD] age, 42.6 [22.9] years) who were attached to these physicians. Most physicians provided between 40% and 80% of care virtually. A higher percentage of the physicians who provided more than 80% of care virtually were 65 years or older, female individuals, and practiced in big cities. Patient comorbidity and morbidity were similar across strata of virtual care use. The mean (SD) number of ED visits was highest among patients whose physicians provided only in-person care (470.3 [1918.8] per 1000 patients) and was lowest among patients of physicians who provided more than 80% to less than 100% of care virtually (242.0 [800.3] per 1000 patients). After adjustment for patient characteristics, patients of physicians with more than 20% of visits delivered virtually had lower rates of ED visits compared with patients of physicians who provided more than 0% to 20% of care virtually (eg, >80% to <100% vs >0%-20% virtual visits in big cities: relative rate, 0.77%; 95% CI, 0.74%-0.81%). This pattern was unchanged across all rurality of practice strata and after adjustment for 2019 ED visit rates. In urban areas, there was a gradient whereby patients of physicians providing the highest level of virtual care had the lowest ED visit rates.

**Conclusions and Relevance:**

Findings of this study show that patients of physicians who provided a higher percentage of virtual care did not have higher ED visit rates compared with patients of physicians who provided the lowest levels of virtual care. The findings refute the hypothesis that family physicians providing more care virtually during the pandemic resulted in higher ED use.

## Key Points

**Question:**

Do patients of family physicians who provide more virtual care have higher emergency department (ED) visit rates?

**Findings:**

In this cross-sectional study of 13 820 family physicians with 12 951 063 patients in Ontario, Canada, we found that patients of physicians who provided a high percentage of virtual care during the first years of the COVID-19 pandemic did not have higher ED visits than patients of physicians who provided the lowest levels of virtual care. This finding remained unchanged after adjusting for patient characteristics.

**Meaning:**

Findings of this study suggest that more virtual care from family physicians during the pandemic did not result in more ED use.

## Teaser

This cross-sectional study examines the association between virtual visits and emergency department visits among patients with a family physician who provides virtual care.

## Introduction

The COVID-19 pandemic prompted the seismic shift in how primary care is delivered in the US. Shortly after the global pandemic was declared in March 2020, the amount of virtual care being provided increased 56-fold, representing more than 70% of all primary care visits in Ontario, Canada.^1^ More than 2 years into the pandemic, virtual care continues to comprise a substantial portion of visits.^2^ Research has found that most patients feel comfortable with virtual care, like its convenience, and want it to continue.^3,4,5,6^ However, there are concerns about the implications of virtual visits for care quality, including patient safety,^7^ equity in access,^8,9^ chronic condition management,^10^ and health care use.^11^

The proportion of primary care delivered virtually is affected by factors such as reimbursement and overhead, patient and clinician access to technology and infrastructure, clinician access to personal protective equipment, clinician health concerns, and patient and clinician preferences.^12,13,14,15,16,17^ There have been concerns that, in some physician practices, the proportion of virtual visits is too high, which may lead to high use in other parts of the health system. However, few studies have tested this hypothesis. One such study found that high practice telehealth use was associated with a small increase in emergency department (ED) visits and hospitalizations for ambulatory care sensitive conditions (ACSC) compared with low practice telehealth use.^11^

In Ontario, Canada, provincial authorities directed family physicians to adopt a virtual-first approach early in the pandemic when COVID-19 case counts were high. Specifically, physicians were required to perform the initial assessment remotely and then see patients in person only if warranted. However, by fall 2021, authorities were concerned that family physicians were providing too much care virtually, contributing to increased ED use.^18,19^ Emergency departments faced long wait times, and there were anecdotal reports of family physicians refusing to see people in person but instead sending them to the ED for a needed examination, contributing to ED strain.^18^ At that time, virtual visits paid the same as in-person visits despite the latter being inherently more costly and riskier for COVID-19 exposure. In October 2021, the Ontario Chief Medical Officer of Health and the College of Physicians and Surgeons of Ontario issued a joint statement encouraging more use of in-person care.^20^ It is unclear, however, whether high use of virtual care is associated with perceived increases in ED visit volumes. We undertook a cross-sectional study using routinely collected data to evaluate the association between the percentage of virtual visits in primary care and the rate of ED visits.

## Methods

Use of data in this study was authorized and did not require research ethics board review under section 45 of the Personal Health Information Protection Act of Ontario, Canada. We followed the Strengthening the Reporting of Observational Studies in Epidemiology (STROBE) reporting guideline.

### Study Design, Context, and Setting

We first examined primary care and ED visit patterns in Ontario, Canada, between January 1, 2019, and October 31, 2021. We then conducted a cross-sectional analysis to analyze the association between the percentage of care delivered virtually by primary care physicians and the ED visit rates among patients between February 1 and October 31, 2021. Both population-based analyses used linked administrative data.

Ontario is Canada’s largest province, with a population of 14.8 million in 2021.^21^ Physician and hospital visits are fully covered by provincial insurance for all permanent residents. More than 80% of the population is formally enrolled with a family physician who practices in a patient enrollment model (PEM) wherein, depending on the model of care, physicians receive between 15% and 70% of payment via capitation, along with some fee-for-service and incentive payments.^22^ Family physicians in a PEM have overall responsibility for meeting the primary care needs of enrolled patients; incentive payments are intended to discourage physicians from sending patients to seek care outside of the enrolling group.^23^ The remaining Ontario population either are unattached to a family physician or see a family physician in a fee-for-service practice. Approximately one-quarter of Ontario residents live in rural communities with fewer than 30 000 people.^24^ Rural EDs are staffed by family physicians and have visit rates that are higher than in urban areas due in part to the limited availability of other after-hours care.^25^

Temporary virtual care billing codes were introduced in Ontario on March 14, 2020, which paid the same amount as an equivalent in-person visit during the study period. Initially, the same billing code was used for both telephone and video visits.

### Study Population

We identified ED visit patterns for all residents in Ontario. For the cross-sectional study, we included family physicians with at least 1 primary care visit claim between February 1 and October 31, 2021, and permanent residents who were alive as of March 31, 2021. We assigned residents to a family physician using enrollment tables from the Ontario Ministry of Health; those who were not enrolled were assigned to the family physician who billed the highest amount for their care according to core primary care fee codes used within the previous 2 years (eAppendix 1 in Supplement 1). We excluded residents whom we could not assign to a family physician or whose family physician had no visit claims during the study period. We categorized physicians according to their practice group either under a traditional fee-for-service system or 1 of the following 3 types of PEMs: enhanced fee-for-service, wherein approximately 80% of income comes from fee-for-service payment; non–team-based capitation, wherein approximately 70% of income comes from capitated payment; and team-based capitation, wherein approximately 70% of income comes from capitated payment and physicians have access to a government-funded interprofessional team.

### Data Sources and Measurement

We used the Ontario Health Insurance Plan (OHIP) Claims Database to count primary care physician visits weekly by type and the National Ambulatory Care Reporting System to assess ED visits weekly (eAppendix 2 in Supplement 1). For context, we included publicly available data on COVID-19 case counts in Ontario from Public Health Ontario.^26^

We identified physician characteristics from the Corporate Provider Database; model of care and panel size from the Primary Care Population data set; and patient age, sex, and postal code from the Registered Persons Database of individuals who had or were eligible for provincial health insurance. Race and ethnicity data are not routinely collected in Ontario and thus were not available in the data holdings. We used postal code data and the 2016 Canadian Census data (from the Postal Code Conversion File) to derive neighborhood-level income quintile. We ascertained rurality from patient and physician postal codes and the Rurality Index of Ontario score.^27^ Patient postal code and the Ontario Marginalization Index^28,29^ were used to assess neighborhood-level material deprivation and ethnic diversity. Registration for OHIP within the past 10 years was a proxy for recent immigration to Ontario. We used the Johns Hopkins Adjusted Clinical Group method (ACG System, version 10) to classify patient comorbidity (via Aggregated Diagnosis Groups) and morbidity (via Resource Utilization Bands).^30,31^

Data sets were linked using unique encoded identifiers and analyzed at ICES, which is an independent, nonprofit research institute whose legal status under Ontario’s health information privacy law allows ICES to collect and analyze health care and demographic data, without consent, for the purpose of health system evaluation and improvement. Data sources are summarized in eAppendix 2 in Supplement 1.

### Statistical Analysis

For each family physician, we calculated the percentage of all visits that were delivered virtually between February 1 and October 31, 2021. We stratified physicians into the following groups a priori by their percentage of virtual care use: 0% (100% in person), more than 0% to 20%, more than 20% to 40%, more than 40% to 60%, more than 60% to 80%, more than 80% to less than 100%, and 100%. We described patient and physician characteristics as of March 31, 2021, within each stratum of virtual care use.

For physicians in a PEM, we examined the variation in virtual care use among physicians in the same practice group and physicians in different practice groups, stratified by type of PEM. For this analysis, the percentage of virtual visits delivered was modeled as a continuous variable for each physician. To understand how much of the total variance in virtual visits was attributable to practice group and PEM type, we calculated an intraclass correlation coefficient (ICC) from a 3-level (patient, physician, and group), intercept-only mixed linear model, with virtual visit rate as the outcome.

We examined the health care use of patients who were enrolled with family physicians in different strata of virtual care use. We compared outcomes across the strata of virtual care use. For each stratum, we assessed the number of ED visits for every patient and calculated the mean number per 1000 patients. We also assessed the following contextual information: (1) the percentage of patients with a primary care visit, (2) the mean number of primary care visits, and (3) the percentage of visits to family physicians. Secondary outcomes included the percentage of patients with ACSC-related hospital admissions, which were derived from the Canadian Institute for Health Information Discharge Abstract Database (eAppendix 3 in Supplement 1), and specialist visits, which were ascertained from physician billings. We examined bivariate associations overall and separately for each stratum of rurality of practice (big cities, small cities, small towns, and rural areas). Next, to understand whether the associations observed in 2021 reflected the associations before the COVID-19 pandemic, we examined the absolute and relative differences in the ED visit rate between the study period (February 1-October 31, 2021) and the corresponding period in 2019 for each stratum of virtual care use.

To adjust for potential confounders, we constructed 2 multivariable negative binomial regression models with a physician-level exposure (percentage of care delivered virtually as a 5-level categorical variable) and a patient-level outcome (the number of ED visits per patient). We used generalized estimating equations to account for the clustering of patients within physicians, with a compound symmetric covariance structure type. In model 1, we adjusted for patient age, sex, neighborhood income quintile, recent OHIP registration, comorbidity and morbidity, and health care use, and we included an interaction term for the exposure and rurality of practice. Model 2 included the same covariates and was further adjusted for the ED visit rate in 2019 to account for potential preexisting patterns. All analyses were performed in SAS Enterprise Guide, version 7.1 (SAS Institute Inc). Details on model selection and fit are provided in eAppendix 4 in Supplement 1. In a sensitivity analysis, we used a published algorithm^32^ to restrict the cohort to comprehensive primary care physicians who were active as of March 31, 2019, the most recent year for which the data for the algorithm were available.

## Results

We analyzed data for 13 820 family physicians (6706 females [48.5%] and 7114 males [51.5%]; mean [SD] age, 50 [13.1] years) who had at least 1 primary care visit claim during the study period and 12 951 063 Ontario residents (6 714 150 females [51.8%] and 6 236 913 males [48.2%]; mean [SD] age, 42.6 [22.9] years) who were attached to these physicians (Table 1; eAppendix 5 in Supplement 1). Most physicians provided between 40% and 80% of care virtually; only 336 physicians delivered 100% of care virtually, while 2606 provided more than 80% but less than 100% of care virtually. Compared with physicians in other virtual care use strata, a higher percentage of the physicians in the more-than-80%-to-less-than-100% stratum were 65 years or older, were female individuals, and practiced in big cities. Most physicians in the 100% stratum worked in a traditional fee-for-service practice and had a panel size of less than 100 patients; more of their patients were recent OHIP registrants and lived in lower-income, ethnically diverse neighborhoods (eAppendix 5 in Supplement 1). Patient comorbidity and morbidity were similar across the virtual care use strata.

**Table 1.** Physician and Practice Characteristics by Percentage of Care Delivered Virtually

Characteristic	Percentage of virtual care, No. (%)							Total, No. (%)
	0	>0-20	>20-40	>40-60	>60-80	>80-<100	100	
No. of physicians^a^	863 (6.2)	1257 (9.1)	1726 (12.5)	2932 (21.2)	4100 (29.7)	2606 (18.9)	336 (2.4)	13 820 (100)
No. of patients attached to these physicians	82 689 (0.6)	771 808 (6.0)	1 633 324 (12.6)	3 229 223 (24.9)	4 657 341 (36.0)	2 511 964 (19.4)	64 714 (0.5)	12 951 063 (100)
Physician age								
≤44 y	356 (41.3)	405 (32.2)	681 (39.5)	1268 (43.2)	1719 (41.9)	918 (35.2)	116 (34.5)	5463 (39.5)
45-64 y	338 (39.2)	590 (46.9)	766 (44.4)	1304 (44.5)	1878 (45.8)	1147 (44.0)	112 (33.3)	6135 (44.4)
65-74 y	117 (13.6)	193 (15.4)	221 (12.8)	297 (10.1)	420 (10.2)	424 (16.3)	75 (22.3)	1747 (12.6)
≥75 y	39 (4.5)	69 (5.5)	58 (3.4)	63 (2.1)	83 (2.0)	117 (4.5)	33 (9.8)	462 (3.3)
Missing data	13 (1.5)	0 (0)	0 (0)	0 (0)	0 (0)	0 (0)	0 (0)	13 (0.1)
Physician sex								
Female	319 (37.0)	415 (33.0)	700 (40.6)	1480 (50.5)	2296 (56.0)	1309 (50.2)	187 (55.7)	6706 (48.5)
Male	544 (63.0)	842 (67.0)	1026 (59.4)	1452 (49.5)	1804 (44.0)	1297 (49.8)	149 (44.3)	7114 (51.5)
Practice group								
PEM: non–team-based capitation	14 (1.6)	148 (11.8)	354 (20.5)	788 (26.9)	1267 (30.9)	570 (21.9)	11 (3.3)	3152 (22.8)
PEM: enhanced fee-for-service	31 (3.6)	194 (15.4)	332 (19.2)	492 (16.8)	923 (22.5)	777 (29.8)	32 (9.5)	2781 (20.1)
PEM: team-based capitation	31 (3.6)	102 (8.1)	377 (21.8)	980 (33.4)	1172 (28.6)	359 (13.8)	12 (3.6)	3033 (21.9)
Traditional fee-for-service	787 (91.2)	813 (64.7)	663 (38.4)	672 (22.9)	738 (18.0)	900 (34.5)	281 (83.6)	4854 (35.1)
Rurality of practice								
Big cities	372 (43.1)	540 (43.0)	683 (39.6)	1179 (40.2)	2128 (51.9)	1510 (57.9)	213 (63.4)	6625 (47.9)
Small cities	182 (21.1)	330 (26.3)	421 (24.4)	784 (26.7)	1169 (28.5)	759 (29.1)	75 (22.3)	3720 (26.9)
Small towns	165 (19.1)	225 (17.9)	353 (20.5)	599 (20.4)	500 (12.2)	221 (8.5)	24 (7.1)	2087 (15.1)
Rural areas	131 (15.2)	162 (12.9)	269 (15.6)	370 (12.6)	303 (7.4)	116 (4.5)	24 (7.1)	1375 (9.9)
Missing data	13 (1.5)	0 (0)	0 (0)	0 (0)	0 (0)	0 (0)	0 (0)	13 (0.1)
Patient panel size								
<100 Patients	744 (86.2)	578 (46.0)	448 (26.0)	467 (15.9)	476 (11.6)	579 (22.2)	238 (70.8)	3530 (25.5)
100-499 Patients	72 (8.3)	230 (18.3)	235 (13.6)	278 (9.5)	363 (8.9)	350 (13.4)	54 (16.1)	1582 (11.4)
500-999 Patients	18 (2.1)	127 (10.1)	261 (15.1)	608 (20.7)	897 (21.9)	493 (18.9)	25 (7.4)	2429 (17.6)
1000-1499 Patients	21 (2.4)	120 (9.5)	356 (20.6)	722 (24.6)	1214 (29.6)	546 (21.0)	9 (2.7)	2988 (21.6)
1500-1999 Patients	≤5 (≤0.6)	85 (6.8)	225 (13.0)	489 (16.7)	697 (17.0)	348 (13.4)	≤5 (≤1.5)	1855 (13.4)
≥2000 Patients	≤5 (≤0.6)	117 (9.3)	201 (11.6)	368 (12.6)	453 (11.0)	290 (11.1)	≤5 (≤1.5)	1436 (10.4)

Abbreviation: PEM, patient enrollment model.

^a^ Including all physicians who had rostered or virtually rostered patients as of March 31, 2021, and had at least 1 primary care visit between February 1 and October 31, 2021. Ontario residents who were not attached to a primary care physician or whose physician did not have visit claims during the study period were excluded.

**Figure 1.**
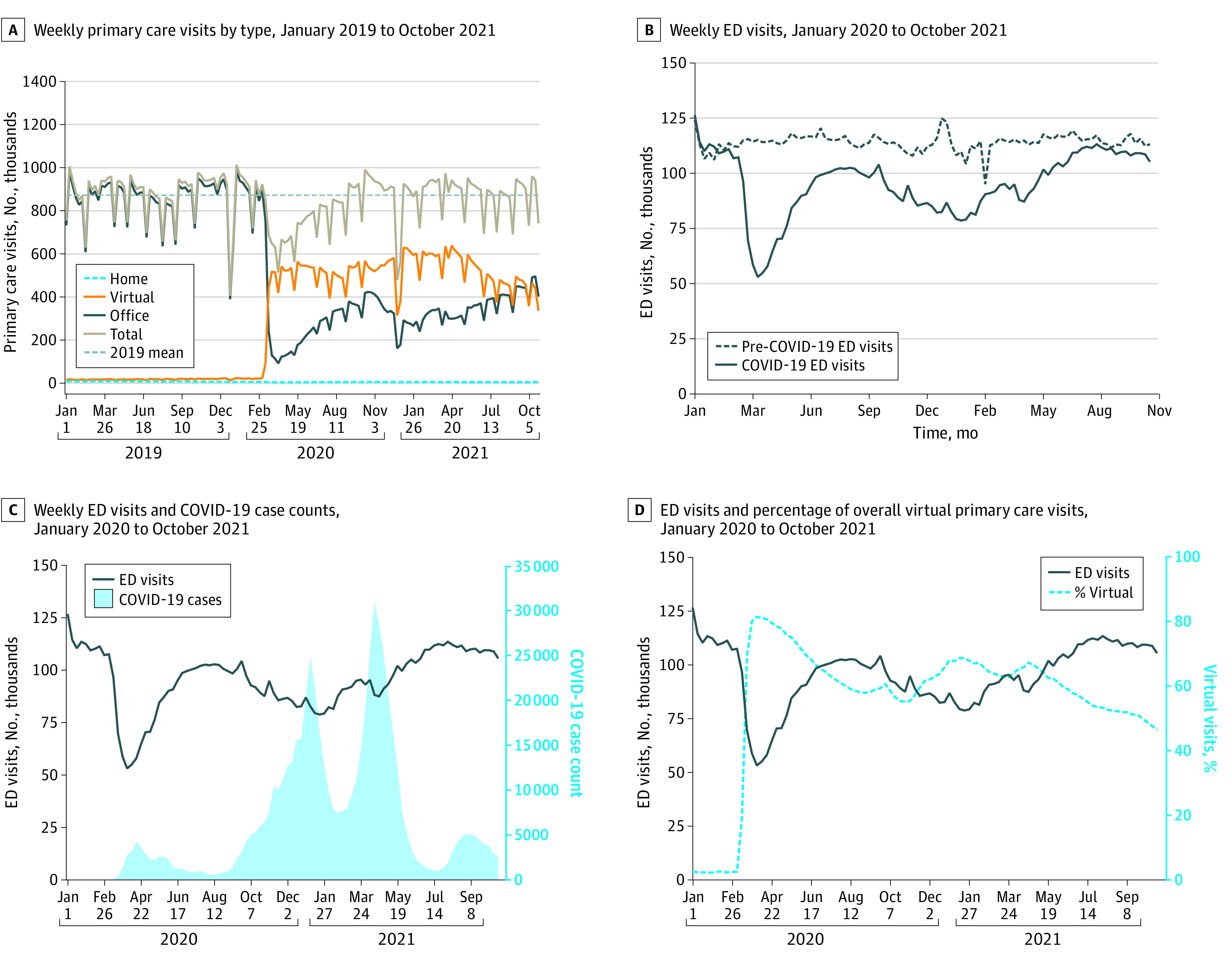
Primary Care Visits, Emergency Department (ED) Visits, and Percentage of Total Primary Care Visits Delivered Virtually From January 2019 to October 2021

Total number of primary care visits decreased at the onset of the COVID-19 pandemic but returned to mean prepandemic levels by fall 2020 (Figure 1A). The percentage of virtual primary care visits peaked in the first 2 weeks of the pandemic at 82% but was 49% by October 2021. The ED visit rates decreased at the start of the pandemic and remained lower than the 2019 volumes throughout the study period (Figure 1B). Patterns in ED visit rates were similar for Canadian Triage and Acuity Scale levels 2, 3, and 4 (eAppendix 6 in Supplement 1), with declines visually corresponding to increases in COVID-19 case numbers (Figure 1C). Periods with a higher percentage of virtual primary care visits visually corresponded to higher COVID-19 case numbers and lower ED visit rates (Figures 1C and D).

**Figure 2.**
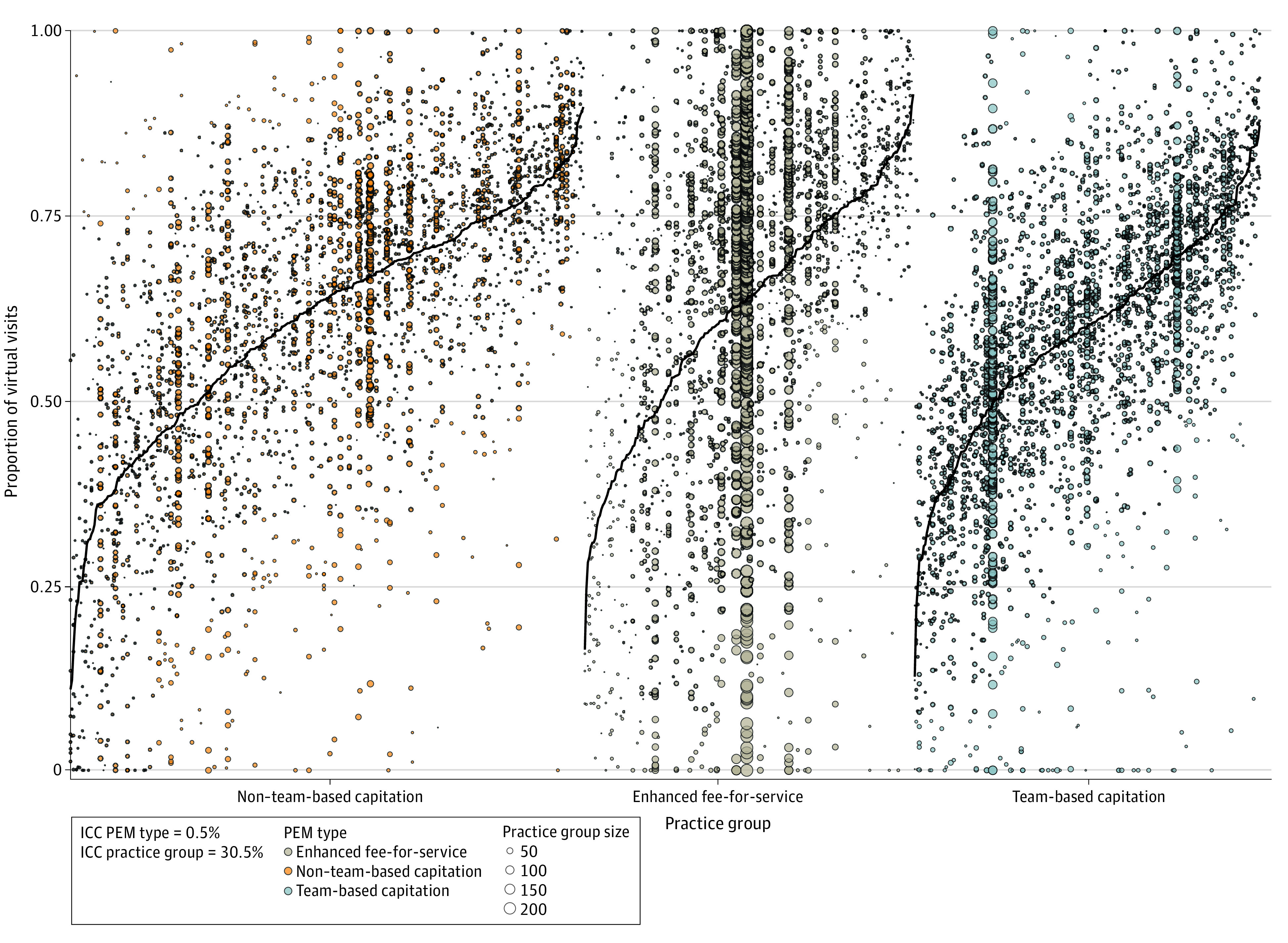
Variation in the Percentage of Virtual Visits Among Physicians in the Same Practice Group and Physicians in Different Practice Groups Stratified by Type of Patient Enrollment Model (PEM) From February to October 2021 Black lines represent the mean ratio for the practice group. Each group can have 3 or more physicians, and each dot represents a physician. Physicians within the same practice group are represented on the same vertical line. An intraclass correlation coefficient (ICC) was calculated using a 3-level, intercept-only mixed linear model to ascertain how much of the total variance in virtual visits was attributable to practice group and PEM type. Variation was not explained by PEM type (ICC, 0.5%), whereas a high percentage of variation was explained by specific practice group (ICC, 30.5%).

Among physicians practicing in a PEM, we found substantial variation in the percentage of virtual care provided by physicians within the same or from different practice groups as shown in Figure 2. The variation was not explained by PEM type (ICC, 0.5%); practice group accounted for almost one-third of the observed variation between all physicians (ICC, 30.5%).

Compared with patients whose physicians provided 40% to 60% of care virtually, patients whose physicians provided more than 80% of care virtually had a higher mean number of primary care visits per patient and lower care continuity. These physicians did not have substantially different referral rates to specialists or hospital admission rates for ACSC (Table 2).

**Table 2.** Patient Health Service Use Stratified by the Rostered Physician’s Percentage of Care Delivered Virtually Between February 1 and October 31, 2021

	Percentage of virtual care, No. (%)							Total, No. (%)
	0	>0-20	>20-40	>40-60	>60-80	>80-<100	100	
No. of patients	82 689 (0.6)	771 808 (6.0)	1 633 324 (12.6)	3 229 223 (24.9)	4 657 341 (36.0)	2 511 964 (19.4)	64 714 (0.5)	12 951 063 (100)
Primary care services								
Any primary care visit	46 285 (56.0)	476 755 (61.8)	1 011 337 (61.9)	2 015 294 (62.4)	2 998 836 (64.4)	1 652 188 (65.8)	40 898 (63.2)	8 241 593 (63.6)
Mean No. of primary care visits per patient (SD)	2.5 (4.9)	2.5 (4.2)	2.4 (3.9)	2.3 (3.7)	2.6 (3.9)	2.9 (4.3)	3.1 (5.34)	2.5 (3.9)
Mean percentage of visits with most responsible family physician (SD)^a^	47.9 (44.8)	66.1 (40.3)	70.0 (38.2)	71.7 (36.7)	71.5 (36.8)	67.9 (38.9)	39.5 (42.5)	70.0 (37.8)
Other health care use								
Any specialist visits	31 481 (38.1)	290 308 (37.6)	621 311 (38.0)	1 251 610 (38.8)	1 855 914 (39.8)	1 012 442 (40.3)	25 745 (39.8)	5 088 811 (39.3)
Ambulatory care–sensitive condition visits	324 (0.4)	2085 (0.3)	4104 (0.3)	7761 (0.2)	9608 (0.2)	4699 (0.2)	114 (0.2)	28 695 (0.2)
ED use, February to October 2019 and 2021								
Total visits per 1000 in 2021, mean (SD)^b^	470.3 (1918.8)	309.8 (1087.0)	295.3 (1036.1)	283.5 (919.7)	253.4 (825.4)	242.0 (800.3)	287.1 (1048.9)	268.9 (903.2)
Total visits per 1000 in 2019, mean (SD)^b^	468.3 (1850.5)	360.6 (1100.1)	346.5 (996.9)	329.0 (985.8)	290.1 (934.2)	272.0 (843.2)	294.0 (891.4)	309.6 (965.4)
Absolute difference, 2021 vs 2019 (95% CI)	2.0 (−14.1 to 18.1)	−50.7 (−54.2 to −47.3)	−51.3 (−53.6 to −49.0)	−45.5 (−47.0 to −44.0)	−36.7 (−37.9 to −35.5)	−30.0 (−31.4 to −28.5)	−6.9 (−16.8 to 3.0)	−40.7 (−41.44 to −40.0)
% Change, 2021 vs 2019 (95% CI)	0.4 (−3.0 to 3.9)	−14.1 (−15.0 to −13.2)	−14.8 (−15.4 to −14.2)	−13.8 (−14.3 to −13.4)	−12.7 (−13.0 to −12.3)	−11.0 (−11.5 to −10.5)	−2.4 (−5.8 to 1.1)	−13.2 (−13.4 to −12.9)

Abbreviation: ED, emergency department.

^a^ Among patients with 2 or more visits between February and October 2021.

^b^ Including only patients whose attached physicians had visit claims in both 2021 and 2019.

The mean (SD) number of ED visits was highest among patients whose physicians provided 0% virtual care (ie, only in-person care) (470.3 [1918.8] per 1000 patients) and was lowest among patients whose physicians were in the more-than-80%-to-less-than-100% virtual care use stratum (242.0 [800.3] per 1000 patients) (Table 2). Patients’ ED use decreased as physicians’ percentage of virtual care delivered increased except among physicians who provided 100% virtual care. This pattern was similar when the analysis was repeated using 3-month segments (eAppendix 7 in Supplement 1). The ED visit rates in 2019 demonstrated a similar pattern. Between 2019 and 2021, there was an overall 13% decrease in the mean (SD) number of ED visits (2019: 309.6 [965.4] per 1000 patients; 2021: 268.9 [903.2] per 1000 patients). Visits decreased across the virtual care use strata except for a slight increase among patients who were attached to physicians who provided 0% virtual care. The absolute and relative decreases were smallest among patients of physicians who provided 100% virtual care, followed by patients whose physicians provided more than 80% to less than 100% virtual care. The crude association between the percentage of care delivered virtually and ED use was consistent by rurality of practice strata (eAppendix 8 in Supplement 1).

**Figure 3.**
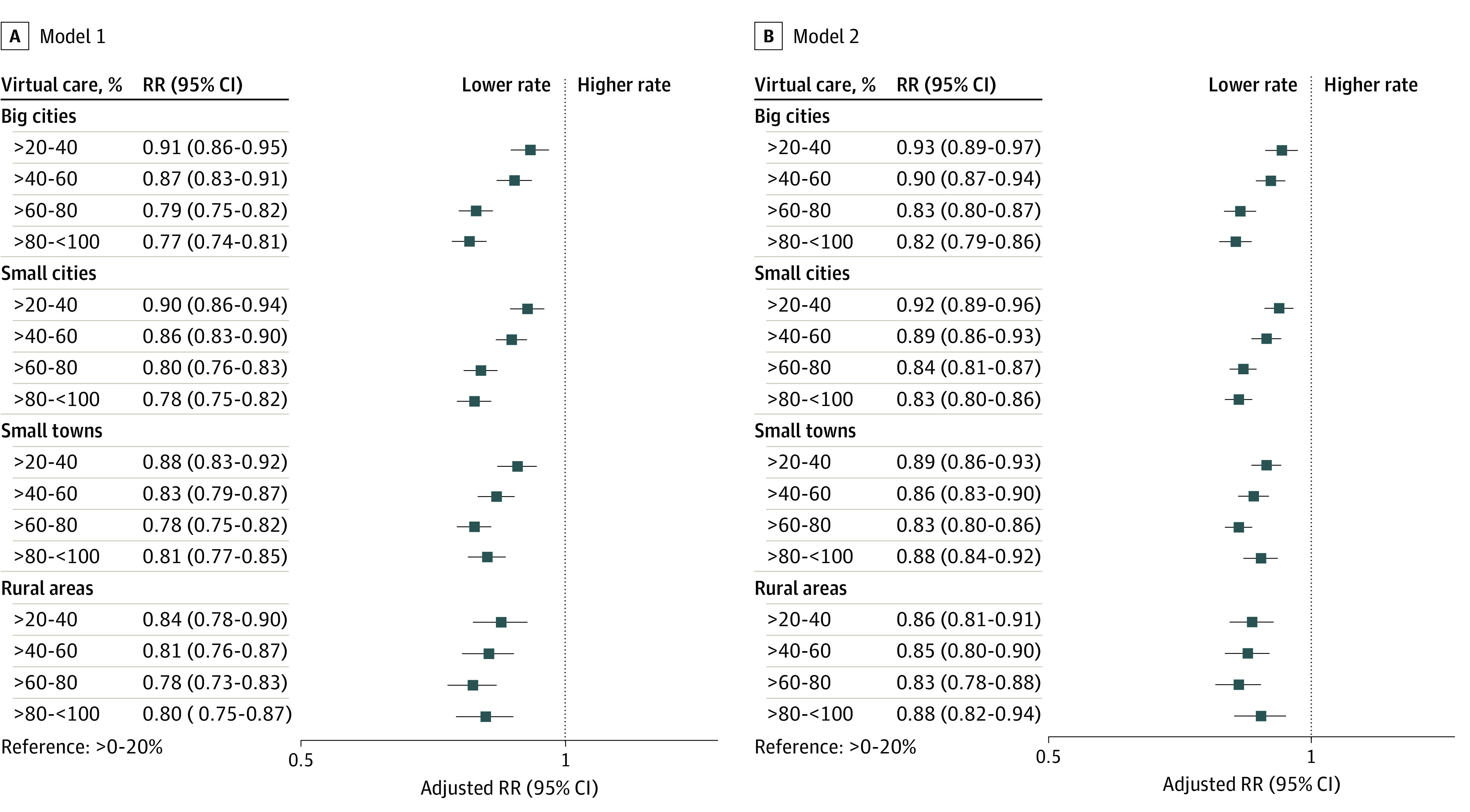
Adjusted Relative Rates (RRs) of Patient Emergency Department Visits by Percentage of Total Visits Delivered Virtually by Family Physicians Models included an interaction term for the exposure and rurality of practice. Model 1 was adjusted for patient age, sex, neighborhood income quintile, recent insurance registration, comorbidity and morbidity, and health care use. Model 2 was adjusted for the same covariates plus ED visit rates in 2019.

Regression modeling excluded patients of physicians who provided 0% (n = 863) or 100% (n = 336) virtual care. After adjustment for patient characteristics, patients with physicians who delivered more than 20% of care virtually had lower rates of ED visits compared with patients of physicians who provided the least virtual care (eg, >80% to <100% vs >0%-20% [reference] virtual visits in big cities: relative rate (RR), 0.77; 95% CI, 0.74-0.81) (Figure 3A). This pattern was consistent across all rurality. These patterns remained after adjusting the model for patient ED visit rates in 2019 (eg, >80% to <100% vs >0%-20% [reference] virtual visits in big cities: RR, 0.82; 95% CI, 0.79-0.86) (Figure 3B). Overall, results were consistent in the sensitivity analysis, which was limited to comprehensive family physicians (n = 9462) (eAppendixes 9-11 in Supplement 1).

## Discussion

The expansion of virtual care during the COVID-19 pandemic opened new modes of access for patients and allowed family physicians to provide care while reducing the risk of COVID-19 transmission. Nonetheless, some policy makers worried that virtual care was being used inappropriately, leading to an increase in ED use. Findings of this study refute this hypothesis. First, we found that population-based ED visit rates were lower in the first year of the pandemic compared with prepandemic levels; increases in ED use seemed to coincide with decreasing COVID-19 cases and did not coincide with increases in virtual primary care visits. Second, we found that, at the population level, patients attached to physicians who provided a high percentage of virtual care did not have a higher rate of ED visits compared with those attached to physicians who provided the lowest levels of virtual care. This finding remained true even after adjusting for patient characteristics, with differences largely following prepandemic patterns.

The ED visit rates in 2021 were lower than in 2019 despite anecdotal reports in the media of strained, overcrowded EDs.^19^ Reasons for this finding are likely multifactorial, including patients who deferred care due to reports of system strain and fear of contracting COVID-19; other data also suggested a decrease in non–COVID-19 viral respiratory infections.^33^ Staffing shortages and strained hospital inpatient capacity due to patients awaiting long-term care and longer length of stay for patients with COVID-19 likely contributed to ED crowding despite lower visit volumes.^34,35,36^

Prior to the pandemic, several studies suggested that virtual care could reduce ED and other hospital use, specifically for rural populations,^37^ older populations,^38^ and after a natural disaster.^39^ Following the start of the pandemic, 1 study found that practices with a high level of virtual care use had a small increase in ACSC-related visits compared with practices with a medium level of virtual care use, but differences disappeared when acute and chronic ACSC were evaluated separately.^11^ Other studies found that virtual care use during the pandemic was higher among patients who had more severe illness, suggesting that virtual care supported care continuity.^1,40^ In the US, virtual care often includes a video component, in contrast to Ontario where most virtual care is delivered by telephone.^3^

### Implications for Policy

The pandemic had a role in the widespread adoption of virtual care, which is here to stay. In Ontario, billing codes for virtual care, introduced on an emergency basis during the pandemic, became permanent in October 2022, with lower remuneration for telephone appointments than for in-person visits. However, 2 years into the pandemic, news reports of EDs being overwhelmed continued, with some speculations that a contributing factor to ED crowding was the inability of patients to see their family physicians in person.^41^ Mixed-methods research is needed to elucidate patients’ experience with accessing their family physicians, reasons for seeking care in the ED, and views on virtual care as well as reasons behind physician-level variation in virtual care provision. Researchers and policy makers should be mindful of different patient subgroups wherein virtual care can either facilitate access (eg, for people in rural areas) or serve as a barrier (eg, for people with language or sensory barriers).

### Limitations

This study has some limitations. We examined virtual care visits using new billing codes, but these codes did not distinguish between video and telephone visits. Billing codes also did not capture other aspects of care, such as email communication with patients or care provided by nonphysician clinicians. We examined a 9-month period of the pandemic, but study findings may not reflect evolving practice patterns. Additionally, the administrative data we used did not explain why there are differences in the amount of care delivered virtually, the appropriateness of virtual care for specific circumstances, and whether virtual care meets patients’ needs.

## Conclusions

In this cross-sectional study, we found that at the population level, patients of physicians who provided a high percentage of virtual care did not have high ED visit rates compared with patients of physicians who provided the lowest levels of virtual care. During the first 18 months of the COVID-19 pandemic, ED visit rates were lower than the prepandemic levels, and periods in which the ED visit rates were highest did not coincide with periods with increased virtual care use. The findings refute the hypothesis that family physicians providing more care virtually during the pandemic resulted in higher ED use.

## Notes

Supplement 1. **eAppendix 1.** Billing Codes Used to Determine the Usual Family Physician for Not Enrolled Patients **eAppendix 2.** Description of Ontario Health Administrative Databases and the Variables Included in This Study **eAppendix 3.** Ambulatory Care Sensitive Condition Related Hospital Admissions Used as Secondary Outcomes to Examine Healthcare Utilization of Patients Attached to Family Physicians Within Each Stratum of Virtual Care Use **eAppendix 4.** Model Selection and Fit **eAppendix 5.** Patient Characteristics by the Percent of Physician Primary Care Provided Virtually, Between February 1 and October 31, 2021, in Ontario, Canada **eAppendix 6.** Weekly Emergency Department Visits by Canadian Triage and Acuity Scale (CTAS) Level, January 2019 to October 2021 **eAppendix 7.** Number of ED Visits per 1000 Patients by Rostered Physician's Ratio of Virtual Visit **eAppendix 8.** Patient Health Service Utilization Stratified by the Attached Physician’s Percent of Care Provided Virtually, Between February 1 and October 2021, in Ontario Canada, by Rurality **eAppendix 9.** Physician and Practice Characteristics by the Percent of Care Provided Virtually, Comprehensive Family Physicians, Between February 1 and October 31, 2021, in Ontario, Canada **eAppendix 10.** Characteristics of Patients Attached to Comprehensive Family Physicians by the Percent of Physician Primary Care Provided Virtually, Between February 1 and October 31, 2021, in Ontario, Canada **eAppendix 11.** Health Service Utilization of Patients Attached to Comprehensive Family Physicians Stratified by the Attached Physician’s Percent of Care Provided Virtually, Between February 1 and October 2021, in Ontario Canada

Supplement 2.
Data Sharing Statement
