# Error in the Role of the Funder/Sponsor Section

**DOI:** 10.1001/jamanetworkopen.2023.18204

**Published:** 2023-05-25

In the Original Investigation titled “Virtual Care and Emergency Department Use During the COVID-19 Pandemic Among Patients of Family Physicians in Ontario, Canada”^1^ published April 25, 2023, the first sentence was omitted from the Role of the Funder/Sponsor section. The first sentence should be “Staff from Ontario Health informed study design.” The article has been corrected.
