# Supplementary material for: Virtual Care and Emergency Department Use During the COVID-19 Pandemic Among Patients of Family Physicians in Ontario, Canada
Source: JAMA Netw Open. 2023 Apr 28;6(4):e239602. doi: 10.1001/jamanetworkopen.2023.9602 (PMC10148195; doi:10.1001/jamanetworkopen.2023.9602)
Supplement: Supplement 1. — eAppendix 1. Billing Codes Used to Determine the Usual Family Physician for Not Enrolled Patients eAppendix 2. Description of Ontario Health Administrative Databases and the Variables Included in This Study eAppendix 3. Ambulatory Care Sensitive Condition Related Hospital Admissions Used as Secondary Outcomes to Examine Healthcare Utilization of Patients Attached to Family Physicians Within Each Stratum of Virtual Care Use eAppendix 4. Model Selection and Fit eAppendix 5. Patient Characteristics by the Percent of Physician Primary Care Provided Virtually, Between February 1 and October 31, 2021, in Ontario, Canada eAppendix 6. Weekly Emergency Department Visits by Canadian Triage and Acuity Scale (CTAS) Level, January 2019 to October 2021 eAppendix 7. Number of ED Visits per 1000 Patients by Rostered Physician's Ratio of Virtual Visit eAppendix 8. Patient Health Service Utilization Stratified by the Attached Physician’s Percent of Care Provided Virtually, Between February 1 and October 2021, in Ontario Canada, by Rurality eAppendix 9. Physician and Practice Characteristics by the Percent of Care Provided Virtually, Comprehensive Family Physicians, Between February 1 and October 31, 2021, in Ontario, Canada eAppendix 10. Characteristics of Patients Attached to Comprehensive Family Physicians by the Percent of Physician Primary Care Provided Virtually, Between February 1 and October 31, 2021, in Ontario, Canada eAppendix 11. Health Service Utilization of Patients Attached to Comprehensive Family Physicians Stratified by the Attached Physician’s Percent of Care Provided Virtually, Between February 1 and October 2021, in Ontario Canada [file jamanetwopen-e239602-s001.pdf]

Supplemental Online Content

Kiran T, Green ME, Strauss R, et al. Virtual care and emergency department use during the COVID-19 pandemic among patients of family physicians in Ontario, Canada. *JAMA Netw Open*. 2023;6(4):e239602. doi:10.1001/jamanetworkopen.2023.9602

eAppendix 1. Billing Codes Used to Determine the Usual Family Physician for Not Enrolled Patients

eAppendix 2. Description of Ontario Health Administrative Databases and the Variables Included in This Study

eAppendix 3. Ambulatory Care Sensitive Condition Related Hospital Admissions Used as Secondary Outcomes to Examine Healthcare Utilization of Patients Attached to Family Physicians Within Each Stratum of Virtual Care Use

eAppendix 4. Model Selection and Fit

eAppendix 5. Patient Characteristics by the Percent of Physician Primary Care Provided Virtually, Between February 1 and October 31, 2021, in Ontario, Canada

eAppendix 6. Weekly Emergency Department Visits by Canadian Triage and Acuity Scale (CTAS) Level, January 2019 to October 2021

eAppendix 7. Number of ED Visits per 1000 Patients by Rostered Physician's Ratio of Virtual Visit

eAppendix 8. Patient Health Service Utilization Stratified by the Attached Physician's Percent of Care Provided Virtually, Between February 1 and October 2021, in Ontario Canada, by Rurality

eAppendix 9. Physician and Practice Characteristics by the Percent of Care Provided Virtually, Comprehensive Family Physicians, Between February 1 and October 31, 2021, in Ontario, Canada

eAppendix 10. Characteristics of Patients Attached to Comprehensive Family Physicians by the Percent of Physician Primary Care Provided Virtually, Between February 1 and October 31, 2021, in Ontario, Canada

eAppendix 11. Health Service Utilization of Patients Attached to Comprehensive Family Physicians Stratified by the Attached Physician's Percent of Care Provided Virtually, Between February 1 and October 2021, in Ontario Canada

This supplemental material has been provided by the authors to give readers additional information about their work.

© 2023 Kiran T et al. *JAMA Network Open.*

eAppendix 1. Billing Codes Used to Determine the Usual Family Physician for Not Enrolled Patients.

Fee code	Description
Primary Care Codes	
A001	MINOR ASSESS.-F.P./G.P.
A002	Family Practice & Practice in General - Enhanced 18 month well baby visit
A003	GEN. ASSESS. -F.P./G.P.
A007	INTERMED.ASSESS/WELL BABY CARE-F.P./G.P./PAED.
A903	GEN/FAM PRACT-PRE-DENTAL/OPER.ASSESS LIMIT 2 PER YEAR/PT
E075	GERIATRIC GENERAL ASSESSMENT PREMIUM
G212	D./T. PROC.-ALLERGY-HYPOSENSITIZATION INJECTION PLUS BASIC
G271	D./T. PROC.-CARDIOV.-ANTICOAGULANT SUPERVISION
G372	D./T. PROC.-INJECTIONS-INTRADERMAL/MUSCULAR ETC. EA. ADD.
G373	D./T. PROC.-INJ. INTRADERMAL/MUSC. BASIC FEE (SHICK TEST)
G365	D./T. PROC.-GYNAECOLOGY-PAPANICOLAOU SMEAR
G538	D&T IMMUNIZATION-WITH VISIT, EACH INJECT.
G539	Injection of unspecified agent - sole reason (first injection)
G590	INFLUENZA AGENT +VISIT
G591	Injection of influenza agent - sole reason
K005	INDIVIDUAL CARE PER 1/2 HR
K013	COUNSELLING-ONE OR MORE PEOPLE-PER 1/2HR.
K017	ANNUAL HEALTH EXAM-CHILD AFT. 2ND BIRTHDAY.
P004	OBS.-PRENATAL CARE-MINOR PRENATAL ASSESS.-SUBSEQ.PRENAT.VIS.
K130	Periodic health visit - adolescent
K131	Periodic health visit - adult aged 18 to 64 inclusive
K132	Periodic health visit - adult 65 years of age and older
K030	DIABETIC MANAGEMENT FEE
K080	Minor assessment - Covid, Virtual
K081	Intermediate assessment - Covid, Virtual
K082	Primary mental health care - Covid, Virtual
Paediatric Codes	
A261	MINOR ASSESS.-PAED.
A268	Paediatrics - Enhanced 18 month well baby visit
K267	ANNUAL HEALTH EXAM-CHILD-AFT. 2ND BIRTHDAY PAED.
K269	ANNUAL HEALTH EXAM-PAEDIATRICS-ADOLESCENT-OFFICE

© 2023 Kiran T et al. *JAMA Network Open.*

eAppendix 2. Description of Ontario health administrative databases and the variables included in this study.

Dataset	Description	Variable(s)
National Ambulatory Care Reporting System (NACRS)	NACRS contains clinical, demographic, and administrative information for all patient visits made to hospital- and community-based ambulatory care centres in Ontario.	- Emergency department visits
Discharge Abstract Database (DAD)	DAD summarizes the hospital discharge information of individuals receiving inpatient care in a non-mental health designated bed. Each record contains demographic, clinical and administrative data regarding the hospitalized individual, as well as comorbidities and procedures received.	- Hospitalizations
Primary Care Population (PCPOP) Dataset	PCPOP captures all individuals in Ontario who are alive and eligible for OHIP coverage at a given point in time. The PCPOP dataset includes basic demographic information as well as Primary Care Rostering, which represents information on which physician/group and family health team (FHT) the patient is enrolled or virtually enrolled with.	- Physician model of care<br>- Physician patient panel size
Ontario Health Insurance Plan (OHIP) Claims Database	OHIP Claims Database contains approved physician claims data from inpatient, outpatient and long-term care settings for physicians in Ontario. Among other information, each record identifies the physician, the patient, the diagnosis responsible for the claim, the service provided and the date of service.	- Primary care physician visits
Postal Code Conversion File (PCCF)	PCCF is prepared by Statistics Canada and links six-character postal codes to Statistics Canada's standard geographic areas for which census data and other statistics are produced, such as measures of rurality and neighbourhood income quintiles.	- Rurality Index of Ontario (RIO)
Registered Persons Database (RPDB)	RPDB provides basic demographic information for those issued an Ontario health insurance number. It also indicates the time periods for which an individual was eligible to receive publicly funded health insurance benefits and provides the best known postal code for each registrant on July 1st of each year.	- Identification of Ontario population<br>- Patient-level characteristics
Corporate Provider Database (CPDB)	CPDB is used to pay OHIP claims. This data contains addresses, registration and program eligibility information about individual health care providers such as physicians, pharmacists, and other practitioners. This data also includes organizations such as provider groups, hospitals, independent health facilities, primary care groups, and alternate payment health care providers.	- Physician-level characteristics

eAppendix 3. Ambulatory Care Sensitive Condition Related Hospital Admissions Used as Secondary Outcomes to Examine Healthcare Utilization of Patients Attached to Family Physicians within Each Stratum of Virtual Care Use.

Most responsible diagnosis	International classification of diseases (ICD)-10-CA[a] code
Asthma	J45
Diabetes	E10.0, E10.1, E10.63, E10.64, E10.9, E11.0, E11.1, E11.63, E11.64, E11.9, E13.0, E13.1, E13.63, E13.64, E13.9, E14.0, E14.1, E14.63, E14.64, E14.9
Congestive heart failure	I50, J81
Chronic obstructive pulmonary disease	J41, J42, J43, J44, J47, with CCI[b] exclusion 1IJ50, 1IJ76, 1HB53, 1HD53, 1HZ53, 1HB55, 1HD55, 1HZ55, 1HZ85, 1HB54, 1HD54

[a] An enhanced version of ICD-10 developed by the Canadian Institute for Health Information (CIHI) for morbidity classification in Canada

[b] Canadian Classification of Health Interventions

eAppendix 4. Model selection and fit

Our outcome, the count of ED visits during a period of time, was moderately overdispersed, so we chose the Negative Binomial model to account for this overdispersion. A multilevel model would have been ideal since the ratio of virtual visits is measured at the physician-level. However, due to the large number of physicians, models with random effects failed to converge. We therefore used Generalized Estimating Equations(GEE) to account for clustering within physicians, with a compound symmetric covariance structure; this would provide results that are very close to a model with random effects.

The scatter plot of predicted value versus observed value did not show a nice diagonal line at the level of each unique combination of all covariates included in the model, however, it did perfectly line up in diagonal when we aggregated the observed number of ED visits and the predicted value at each combined level of rural index and ratio of virtual visit, which are the two key factors we were interested in.

In order to reduce the effect of some high number of ED visits, we dichotomized the volume of ED visits and ran a modified Poisson regression to compare with the results to those obtained using the Negative Binomial model. Although the outcome for the two models are not exactly the same, results from the modified Poisson model showed the same pattern as those from the Negative Binomial model, i.e. the risk of visiting ED decreased as the ratio of virtual visit increased, although the difference between virtual ratio groups became smaller.

© 2023 Kiran T et al. *JAMA Network Open.*

eAppendix 5. Patient Characteristics by the Percent of Physician Primary Care Provided Virtually, between February 1 and October 31, 2021, in Ontario, Canada.

	Percent Virtual Care of Primary Care Physician							
**Characteristic, n (%)**	**0%**	**>0-20%**	**>20-40%**	**>40-60%**	**>60-80%**	**>80-<100**	**100%**	**Total**
**No.**	N=82,689	N=771,808	N=1,633,324	N=3,229,223	N=4,657,341	N=2,511,964	N=64,714	N=12,951,063
**Age**								
≤18	11,599 (14.0)	136,211 (17.6)	307,286 (18.8)	615,844 (19.1)	852,541 (18.3)	412,339 (16.4)	10,696 (16.5)	2,346,516 (18.1)
19-29	14,175 (17.1)	107,928 (14.0)	222,336 (13.6)	423,268 (13.1)	627,804 (13.5)	361,294 (14.4)	10,954 (16.9)	1,767,759 (13.6)
30-44	18,044 (21.8)	154,324 (20.0)	319,894 (19.6)	637,641 (19.7)	967,437 (20.8)	551,582 (22.0)	15,833 (24.5)	2,664,755 (20.6)
45-64	21,457 (25.9)	217,806 (28.2)	449,237 (27.5)	898,003 (27.8)	1,316,447 (28.3)	730,943 (29.1)	17,145 (26.5)	3,651,038 (28.2)
65-74	8,724 (10.6)	87,499 (11.3)	185,928 (11.4)	370,765 (11.5)	508,476 (10.9)	263,437 (10.5)	5,938 (9.2)	1,430,767 (11.0)
≥75	8,690 (10.5)	68,040 (8.8)	148,643 (9.1)	283,702 (8.8)	384,636 (8.3)	192,369 (7.7)	4,148 (6.4)	1,090,228 (8.4)
**Sex**								
Female	37,134 (44.9)	375,362 (48.6)	820,252 (50.2)	1,669,636 (51.7)	2,470,394 (53.0)	1,307,088 (52.0)	34,284 (53.0)	6,714,150 (51.8)
Male	45,555 (55.1)	396,446 (51.4)	813,072 (49.8)	1,559,587 (48.3)	2,186,947 (47.0)	1,204,876 (48.0)	30,430 (47.0)	6,236,913 (48.2)
**Neighbourhood-level income quintile**								
1 (lowest)	21,468 (26.0)	188,496 (24.4)	340,108 (20.8)	580,649 (18.0)	782,862 (16.8)	473,926 (18.9)	18,230 (28.2)	2,405,739 (18.6)
2	17,266 (20.9)	164,458 (21.3)	332,357 (20.3)	617,686 (19.1)	870,823 (18.7)	494,764 (19.7)	13,576 (21.0)	2,510,930 (19.4)
3	15,646 (18.9)	155,461 (20.1)	341,044 (20.9)	666,171 (20.6)	942,534 (20.2)	510,991 (20.3)	12,547 (19.4)	2,644,394 (20.4)
4	14,969 (18.1)	141,287 (18.3)	324,103 (19.8)	685,002 (21.2)	993,671 (21.3)	515,099 (20.5)	10,214 (15.8)	2,684,345 (20.7)
5 (highest)	12,634 (15.3)	119,845 (15.5)	291,727 (17.9)	671,129 (20.8)	1,055,796 (22.7)	510,846 (20.3)	9,958 (15.4)	2,671,935 (20.6)
Missing	706 (0.9)	2,261 (0.3)	3,985 (0.2)	8,586 (0.3)	11,655 (0.3)	6,338 (0.3)	189 (0.3)	33,720 (0.3)
**Material Deprivation quintile**								
1 (least deprived)	15,355 (18.6)	126,998 (16.5)	306,477 (18.8)	738,019 (22.9)	1,234,895 (26.5)	609,098 (24.2)	13,683 (21.1)	3,044,525 (23.5)
2	14,157 (17.1)	142,477 (18.5)	330,767 (20.3)	698,625 (21.6)	1,028,992 (22.1)	538,541 (21.4)	10,845 (16.8)	2,764,404 (21.3)
3	14,556 (17.6)	143,940 (18.6)	328,007 (20.1)	620,901 (19.2)	871,073 (18.7)	475,116 (18.9)	11,627 (18.0)	2,465,220 (19.0)
4	15,894 (19.2)	157,907 (20.5)	324,888 (19.9)	583,408 (18.1)	765,832 (16.4)	443,258 (17.6)	12,155 (18.8)	2,303,342 (17.8)
5 (most deprived)	19,541 (23.6)	186,363 (24.1)	327,345 (20.0)	559,404 (17.3)	728,276 (15.6)	433,939 (17.3)	15,934 (24.6)	2,270,802 (17.5)
Missing	3,186 (3.9)	14,123 (1.8)	15,840 (1.0)	28,866 (0.9)	28,273 (0.6)	12,012 (0.5)	470 (0.7)	102,770 (0.8)
**Ethnic Diversity quintile**								
1 (least diverse)	12,908 (15.6)	142,811 (18.5)	350,356 (21.5)	641,513 (19.9)	618,362 (13.3)	202,198 (8.0)	6,382 (9.9)	1,974,530 (15.2)
2	12,470 (15.1)	136,906 (17.7)	322,545 (19.7)	642,478 (19.9)	726,978 (15.6)	274,019 (10.9)	5,966 (9.2)	2,121,362 (16.4)
3	13,694 (16.6)	127,214 (16.5)	271,540 (16.6)	602,331 (18.7)	864,782 (18.6)	408,716 (16.3)	9,656 (14.9)	2,297,933 (17.7)
4	16,790 (20.3)	144,724 (18.8)	276,943 (17.0)	610,992 (18.9)	1,060,728 (22.8)	599,754 (23.9)	15,649 (24.2)	2,725,580 (21.0)
5 (most diverse)	23,641 (28.6)	206,030 (26.7)	396,100 (24.3)	703,043 (21.8)	1,358,218 (29.2)	1,015,265 (40.4)	26,591 (41.1)	3,728,888 (28.8)

© 2023 Kiran T et al. *JAMA Network Open.*

Missing	3,186 (3.9)	14,123 (1.8)	15,840 (1.0)	28,866 (0.9)	28,273 (0.6)	12,012 (0.5)	470 (0.7)	102,770 (0.8)
**Recent Registrant (<10 years)**								
Yes	7,765 (9.4)	72,718 (9.4)	144,967 (8.9)	233,795 (7.2)	403,698 (8.7)	267,726 (10.7)	8,443 (13.0)	1,139,112 (8.8)
No	68,636 (83.0)	624,350 (80.9)	1,318,635 (80.7)	2,652,901 (82.2)	3,790,998 (81.4)	2,030,442 (80.8)	50,453 (78.0)	10,536,415 (81.4)
Missing	6,288 (7.6)	74,740 (9.7)	169,722 (10.4)	342,527 (10.6)	462,645 (9.9)	213,796 (8.5)	5,818 (9.0)	1,275,536 (9.8)
**Morbidity (Resource Utilization Band)**								
0 (non-user)	4,328 (5.2)	59,639 (7.7)	130,063 (8.0)	271,999 (8.4)	379,715 (8.2)	201,771 (8.0)	3,914 (6.0)	1,051,429 (8.1)
1	5,217 (6.3)	51,449 (6.7)	107,877 (6.6)	204,767 (6.3)	279,644 (6.0)	144,922 (5.8)	3,927 (6.1)	797,803 (6.2)
2	17,557 (21.2)	166,766 (21.6)	354,856 (21.7)	687,322 (21.3)	969,771 (20.8)	507,882 (20.2)	13,855 (21.4)	2,718,009 (21.0)
3	38,020 (46.0)	358,321 (46.4)	752,307 (46.1)	1,484,831 (46.0)	2,192,581 (47.1)	1,209,498 (48.1)	31,395 (48.5)	6,066,953 (46.8)
4	11,435 (13.8)	95,981 (12.4)	205,958 (12.6)	415,068 (12.9)	608,355 (13.1)	329,302 (13.1)	8,585 (13.3)	1,674,684 (12.9)
5 (highest user)	6,132 (7.4)	39,652 (5.1)	82,263 (5.0)	165,236 (5.1)	227,275 (4.9)	118,589 (4.7)	3,038 (4.7)	642,185 (5.0)
**Co-Morbidity (Adjusted Diagnoses Groups)**								
0	4,347 (5.3)	59,866 (7.8)	130,708 (8.0)	273,501 (8.5)	381,607 (8.2)	202,442 (8.1)	3,924 (6.1)	1,056,395 (8.2)
1-4	44,431 (53.7)	396,784 (51.4)	839,609 (51.4)	1,627,346 (50.4)	2,284,776 (49.1)	1,202,766 (47.9)	32,437 (50.1)	6,428,149 (49.6)
5-9	26,680 (32.3)	255,438 (33.1)	539,600 (33.0)	1,081,818 (33.5)	1,615,004 (34.7)	890,759 (35.5)	22,758 (35.2)	4,432,057 (34.2)
≥ 10	7,231 (8.7)	59,720 (7.7)	123,407 (7.6)	246,558 (7.6)	375,954 (8.1)	215,997 (8.6)	5,595 (8.6)	1,034,462 (8.0)
**Chronic Conditions**								
Hypertension	20,185 (24.4)	187,063 (24.2)	385,037 (23.6)	737,439 (22.8)	1,025,070 (22.0)	550,637 (21.9)	13,168 (20.3)	2,918,599 (22.5)
DM	10,862 (13.1)	95,571 (12.4)	192,845 (11.8)	359,838 (11.1)	503,761 (10.8)	286,148 (11.4)	6,986 (10.8)	1,456,011 (11.2)
CHF	2,983 (3.6)	16,652 (2.2)	35,249 (2.2)	66,556 (2.1)	84,626 (1.8)	41,660 (1.7)	983 (1.5)	248,709 (1.9)
AMI	1,120 (1.4)	9,454 (1.2)	20,156 (1.2)	38,179 (1.2)	47,613 (1.0)	23,262 (0.9)	493 (0.8)	140,277 (1.1)
Asthma	12,831 (15.5)	118,029 (15.3)	248,568 (15.2)	490,870 (15.2)	726,260 (15.6)	395,342 (15.7)	10,339 (16.0)	2,002,239 (15.5)
COPD	6,148 (7.4)	57,388 (7.4)	124,824 (7.6)	232,495 (7.2)	284,035 (6.1)	139,636 (5.6)	3,539 (5.5)	848,065 (6.5)
Mental health	22,285 (27.0)	157,060 (20.3)	335,569 (20.5)	663,481 (20.5)	994,442 (21.4)	549,442 (21.9)	16,756 (25.9)	2,739,035 (21.1)
**Rurality**								
Big cities	36,934 (44.7)	298,760 (38.7)	545,498 (33.4)	1,122,929 (34.8)	2,033,696 (43.7)	1,259,491 (50.1)	37,770 (58.4)	5,335,078 (41.2)
Small cities	21,951 (26.5)	221,397 (28.7)	444,488 (27.2)	955,035 (29.6)	1,534,725 (33.0)	867,594 (34.5)	16,362 (25.3)	4,061,552 (31.4)
Small towns	13,144 (15.9)	171,619 (22.2)	428,365 (26.2)	809,967 (25.1)	802,412 (17.2)	298,358 (11.9)	4,845 (7.5)	2,528,710 (19.5)
Rural	10,660 (12.9)	80,032 (10.4)	214,973 (13.2)	341,292 (10.6)	286,508 (6.2)	86,521 (3.4)	5,737 (8.9)	1,025,723 (7.9)

© 2023 Kiran T et al. *JAMA Network Open.*

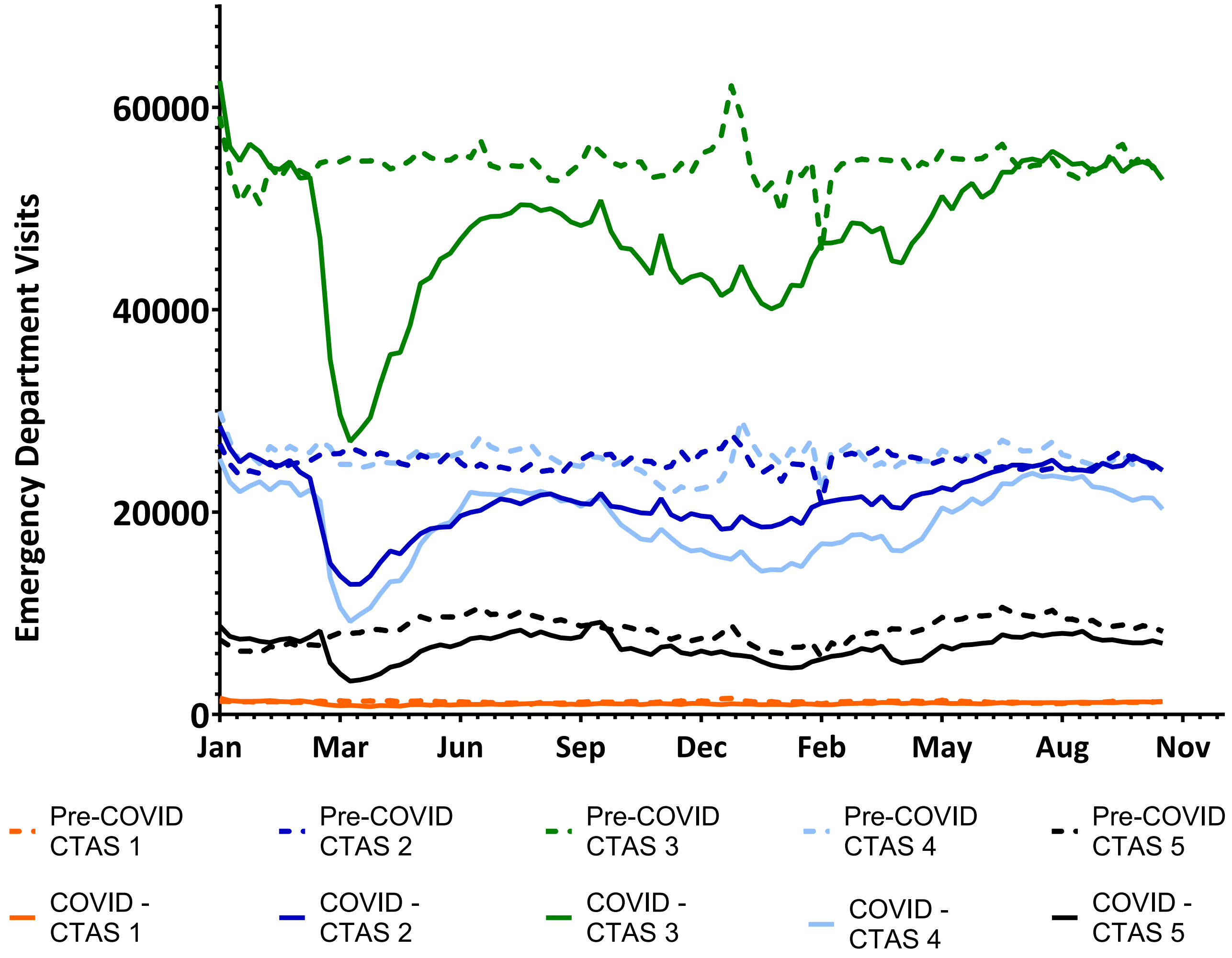

eAppendix 6. Weekly Emergency Department Visits by Canadian Triage and Acuity Scale (CTAS) Level, January 2019 to October 2021.

© 2023 Kiran T et al. *JAMA Network Open.*

eAppendix 7. Number of ED visits per 1000 patients by rostered physician's ratio of virtual visit

VARIABLE	VALUE	0	>0-0.2	>0.2-0.4	>0.4-0.6	>0.6-0.8	>0.8-<1	1	TOTAL
**Feb 1 to Apr 30, 2021**									
Number of physicians		956	900	1217	2058	3848	3921	602	13502
Number of patients		N=111,463	N=557,348	N=1,123,463	N=2,234,969	N=4,402,614	N=4,253,334	N=164,977	N=12,848,168
Number of ED visits per 1000 pts	Mean ± SD	150.72 ± 827.77	91.49 ± 457.37	86.30 ± 419.58	83.45 ± 416.98	79.12 ± 402.39	70.49 ± 362.80	81.76 ± 425.21	78.83 ± 402.64
**May 1 to July 31, 2021**									
Number of physicians		976	1140	1555	2592	3813	2779	527	13382
Number of patients		N=128,402	N=766,321	N=1,539,644	N=2,865,554	N=4,425,467	N=2,908,046	N=139,580	N=12,773,014
Number of ED visits per 1000 pts	Mean ± SD	151.43 ± 754.16	107.66 ± 511.62	102.54 ± 476.90	99.31 ± 443.25	89.99 ± 410.01	85.12 ± 396.26	95.18 ± 413.77	94.22 ± 434.58
**Aug 1 to Oct 31, 2021**									
Number of physicians		1080	1730	2433	3138	2930	1410	426	13147
Number of patients		N=169,517	N=1,412,917	N=2,633,551	N=3,582,994	N=3,380,681	N=1,354,990	N=99,343	N=12,633,993
Number of ED visits per 1000 pts	Mean ± SD	147.85 ± 686.71	113.46 ± 518.62	106.79 ± 465.75	97.44 ± 430.29	89.90 ± 399.99	91.46 ± 409.19	104.30 ± 441.07	99.25 ± 443.31

© 2023 Kiran T et al. *JAMA Network Open.*

eAppendix 8. Patient Health Service Utilization Stratified by the Attached Physician's Percent of Care Provided Virtually, between February 1 and October 2021, in Ontario Canada, by Rurality.

Physician % Virtual Care		0%	>0-20%	>20-40%	>40-60%	>60-80%	>80-<100%	100%	Total
RIO 0	**No. Patients**	N=36,934	N=298,760	N=545,498	N=1,122,929	N=2,033,696	N=1,259,491	N=37,770	N=5,335,078
	**Primary Care Services**								
	Any primary care visit, n (%)	21,703 (58.8)	193,025 (64.6)	356,675 (65.4)	728,409 (64.9)	1,332,732 (65.5)	828,922 (65.8)	24,402 (64.6)	3,485,868 (65.3)
	Number of primary care visits per patient, mean ± SD	2.7 ± 5.1	2.8 ± 4.6	2.8 ± 4.3	2.6 ± 4.0	2.7 ± 4.0	2.9 ± 4.3	3.2 ± 5.3	2.7 ± 4.1
	Percent of visits with usual family physician[a], mean ± SD	52.6 ± 44.6	64.3 ± 41.0	68.1 ± 39.3	70.6 ± 37.5	70.7 ± 37.3	68.0 ± 39.0	45.5 ± 43.0	69.1 ± 38.4
	**Other Health Care Use**								
	Any specialist visits, n (%)	14,756 (40.0)	115,206 (38.6)	211,626 (38.8)	446,582 (39.8)	833,316 (41.0)	519,624 (41.3)	15,286 (40.5)	2,156,396 (40.4)
	Ambulatory care-sensitive condition visits, n (%)	139 (0.4)	766 (0.3)	1,250 (0.2)	2,606 (0.2)	4,027 (0.2)	2,430 (0.2)	67 (0.2)	11,285 (0.2)
	**Emergency Department Utilization, February to October 2019/2021**								
	Total visits per 1,000, 2021, mean ± SD[b]	393.3 ± 1914.7	252.3 ± 933.9	242.0 ± 1120.6	240.6 ± 853.4	223.0 ± 776.2	221.7 ± 760.2	245.2 ± 795.7	231.4 ± 852.9
	Total visits per 1,000, 2019, mean ± SD[b]	414.3 ± 2018.2	293.4 ± 937.8	279.9 ± 906.7	275.8 ± 883.0	256.5 ± 855.4	251.0 ± 798.4	257.3 ± 782.6	265.5 ± 879.7
	Absolute difference 2021 vs 2019 (95% CI)	-21.1 (-47.0, 4.9)	-41.0 (-45.8, -36.3)	-37.9 (-41.8, -33.9)	-35.2 (-37.6, -32.9)	-33.5 (-35.1, -31.9)	-29.3 (-31.2, -27.3)	-12.1 (-22.8, -1.3)	-34.1 (-35.2, -33.1)
	Percent change 2021 vs 2019 (95% CI)	-5.1 (-11.1, 1.1)	-14.0 (-15.5, -12.5)	-13.5 (-14.9, -12.2)	-12.8 (-13.6, -12.0)	-13.1 (-13.7, -12.5)	-11.7 (-12.4, -10.9)	-4.7 (-8.8, -0.6)	-12.9 (-13.2, -12.5)
RIO 1-9	**No. Patients**	N=21,951	N=221,397	N=444,488	N=955,035	N=1,534,725	N=867,594	N=16,362	N=4,061,552
	**Primary Care Services**								
	Any primary care visit, n (%)	12,886 (58.7)	145,329 (65.6)	290,727 (65.4)	622,460 (65.2)	1,023,111 (66.7)	588,907 (67.9)	10,546 (64.5)	2,693,966 (66.3)
	Number of primary care visits per patient, mean ± SD	2.5 ± 4.4	2.7 ± 4.2	2.7 ± 4.1	2.6 ± 3.9	2.8 ± 4.1	3.1 ± 4.5	3.4 ± 6.0	2.8 ± 4.1
	Percent of visits with usual family physician[a], mean ± SD	45.7 ± 44.6	64.5 ± 40.7	66.2 ± 39.3	69.2 ± 37.6	70.9 ± 37.0	67.5 ± 38.8	32.4 ± 41.1	68.6 ± 38.2
	**Other Health Care Use**								
	Any specialist visits, n (%)	8,120 (37.0)	83,129 (37.5)	169,962 (38.2)	371,052 (38.9)	601,803 (39.2)	341,899 (39.4)	6,405 (39.1)	1,582,370 (39.0)

© 2023 Kiran T et al. *JAMA Network Open.*

	Ambulatory care-sensitive condition visits, n (%)	70 (0.3)	517 (0.2)	953 (0.2)	1,963 (0.2)	2,723 (0.2)	1,274 (0.1)	23 (0.1)	7,523 (0.2)
	**Emergency Department Utilization, February to October 2019/2021**								
	Total visits per 1,000, 2021, mean ± SD[b]	356.5 ± 1450.7	257.3 ± 1061.1	242.9 ± 808.5	239.3 ± 811.4	225.2 ± 754.6	219.8 ± 731.0	285.2 ± 1438.8	232.0 ± 797.6
	Total visits per 1,000, 2019, mean ± SD[b]	350.3 ± 1827.1	282.8 ± 944.2	269.6 ± 808.5	268.4 ± 822.5	250.8 ± 785.1	247.8 ± 800.2	281.5 ± 911.3	259.1 ± 825.2
	Absolute difference 2021 vs 2019	6.3 (-22.5, 35.0)	-25.5 (-31.5, -19.4)	-26.7 (-30.2, -23.2)	-29.1 (-31.4, -26.7)	-25.6 (-27.3, -23.8)	-28.0 (-30.3, -25.6)	3.7 (-18.1, 25.5)	-27.2 (-28.3, -26.0)
	Percent change 2021 vs 2019	1.8 (-6.0, 9.8)	-9.0 (-11.1, -6.9)	-9.9 (-11.1, -8.7)	-10.8 (-11.7, -10.0)	-10.2 (-10.9, -9.5)	-11.3 (-12.2, -10.4)	1.3 (-7.5, 10.2)	-10.5 (-10.9, -10.1)
**RIO 10-39**	**No. Patients**	N=13,144	N=171,619	N=428,365	N=809,967	N=802,412	N=298,358	N=4,845	N=2,528,710
	**Primary Care Services**								
	Any primary care visit, n (%)	6,697 (51.0)	96,813 (56.4)	250,322 (58.4)	478,119 (59.0)	486,813 (60.7)	184,204 (61.7)	2,937 (60.6)	1,505,905 (59.6)
	Number of primary care visits per patient, mean ± SD	2.6 ± 5.6	1.9 ± 3.8	1.9 ± 3.3	1.9 ± 3.1	2.1 ± 3.3	2.4 ± 4.1	2.5 ± 4.7	2.0 ± 3.4
	Percent of visits with usual family physician[a], mean ± SD	42.0 ± 45.3	70.9 ± 38.3	75.9 ± 34.9	75.7 ± 34.4	74.4 ± 35.1	68.1 ± 38.7	25.5 ± 36.3	73.7 ± 35.8
	**Other Health Care Use**								
	Any specialist visits, n (%)	4,973 (37.8)	65,808 (38.3)	165,307 (38.6)	312,887 (38.6)	316,486 (39.4)	119,127 (39.9)	1,850 (38.2)	986,438 (39.0)
	Ambulatory care-sensitive condition visits, n (%)	53 (0.4)	558 (0.3)	1,262 (0.3)	2,201 (0.3)	2,043 (0.3)	730 (0.2)	10 (0.2)	6,857 (0.3)
	**Emergency Department Utilization, February to October 2019/2021**								
	Total visits per 1,000, 2021, mean ± SD[b]	564.2 ± 1780.3	371.3 ± 1104.3	334.3 ± 924.4	321.7 ± 924.7	313.2 ± 884.5	326.9 ± 931.4	389.6 ± 970.2	326.6 ± 933.5
	Total visits per 1,000, 2019, mean ± SD[b]	510.7 ± 1472.8	433.2 ± 1147.6	398.7 ± 1018.9	375.9 ± 974.9	360.9 ± 940.5	353.8 ± 944.1	389.1 ± 1125.1	378.6 ± 991.7
	Absolute difference 2021 vs 2019 (95% CI)	53.5 (21.6, 85.5)	-61.9 (-69.5, -54.3)	-64.5 (-68.7, -60.2)	-54.1 (-57.2, -51.1)	-47.8 (-50.7, -44.8)	-26.8 (-31.7, -21.9)	0.5 (-41.3, 42.3)	-52.0 (-53.8, -50.3)
	Percent change 2021 vs 2019	10.5 (3.6, 17.5)	-14.3 (-15.9, -12.7)	-16.2 (-17.1, -15.2)	-14.4 (-15.2, -13.7)	-13.2 (-14.0, -12.5)	-7.6 (-8.9, -6.3)	0.13 (-9.9, 10.6)	-13.7 (-14.2, -13.3)
**RIO 40+**	**No. Patients**	N=10,660	N=80,032	N=214,973	N=341,292	N=286,508	N=86,521	N=5,737	N=1,025,723
	**Primary Care Services**								
	Any primary care visit, n (%)	4,999 (46.9)	41,588 (52.0)	113,613 (52.8)	186,306 (54.6)	156,180 (54.5)	50,155 (58.0)	3,013 (52.5)	555,854 (54.2)

© 2023 Kiran T et al. *JAMA Network Open.*

Number of primary care visits per patient, mean ± SD	1.9 ± 4.4	1.7 ± 3.2	1.6 ± 2.9	1.7 ± 2.8	1.8 ± 3.0	2.3 ± 4.1	1.9 ± 4.1	1.7 ± 3.1
Percent of visits with usual family physician[a], mean ± SD	40.6 ± 42.8	70.7 ± 38.6	74.9 ± 35.5	75.1 ± 34.7	74.9 ± 35.0	70.5 ± 38.8	25.4 ± 38.5	73.7 ± 36.08
**Other Health Care Use**								
Any specialist visits, n (%)	3,632 (34.1)	26,165 (32.7)	74,416 (34.6)	121,089 (35.5)	104,309 (36.4)	31,792 (36.7)	2,204 (38.4)	363,607 (35.4)
Ambulatory care-sensitive condition visits, n (%)	62 (0.6)	244 (0.3)	639 (0.3)	991 (0.3)	815 (0.3)	265 (0.3)	14 (0.2)	3,030 (0.3)
**Emergency Department Utilization, February to October 2019/2021**								
Total visits per 1,000, 2021, mean ± SD[b]	872.8 ± 2751.1	539.5 ± 1532.7	459.9 ± 1369.8	461.6 ± 1304.3	456.6 ± 1227.1	465.8 ± 1319.2	567.1 ± 1264.2	471.0 ± 1341.4
Total visits per 1,000, 2019, mean ± SD[b]	737.3 ± 1896.7	667.7 ± 1716.2	570.5 ± 1401.4	569.8 ± 1556.9	548.9 ± 1772.0	530.2 ± 1312.4	570.1 ± 1224.3	572.6 ± 1589.5
Absolute difference 2021 vs 2019	135.5 (83.2, 187.8)	-128.2 (-144.3, -112.0)	-110.6 (-119.1, -102.0)	-108.2 (-115.2, -101.1)	-92.4 (-100.5, -84.2)	-64.5 (-76.9, -52.1)	-3.0 (-53.7, 47.8)	-101.6 (-105.8, -97.5)
Percent change 2021 vs 2019	18.4 (10.1, 26.8)	-19.2 (-21.4, -17.0)	-19.4 (-20.7, -18.0)	-19.0 (-20.1, -17.9)	-16.8 (-18.1, -15.5)	-12.2 (-14.4, -9.9)	-0.5 (-9.3, 8.6)	-17.8 (-18.4, -17.1)

[a] Among patients with 2 or more visits between February and October 2021

[b] This includes only patients whose attached physicians had claims in both 2021 and 2019.

Abbreviations: SD, standard deviation; CI, confidence interval

© 2023 Kiran T et al. *JAMA Network Open.*

eAppendix 9. Physician and Practice Characteristics by the Percent of Care Provided Virtually, Comprehensive Family Physicians, between February 1 and October 31, 2021, in Ontario, Canada.

**Physician % Virtual Care**	**0%**	**>0-20%**	**>20-40%**	**>40-60%**	**>60-80%**	**>80-<100%**	**100%**	**Total**
**No. Physicians**	N=117	N=544	N=1,118	N=2,265	N=3,432	N=1,881	N=105	N=9,462
**No. Patients**	N=46,747	N=661,761	N=1,476,716	N=2,966,294	N=4,422,486	N=2,386,349	N=51,668	N=12,125,959
**Physician Age**								
≤44	33 (28.2)	114 (21.0)	378 (33.8)	875 (38.6)	1,319 (38.4)	599 (31.8)	43 (41.0)	3,361 (35.5)
45-64	34 (29.1)	258 (47.4)	512 (45.8)	1,076 (47.5)	1,674 (48.8)	862 (45.8)	30 (28.6)	4,446 (47.0)
65-74	37 (31.6)	124 (22.8)	179 (16.0)	256 (11.3)	369 (10.8)	335 (17.8)	23 (21.9)	1,323 (14.0)
≥75	13 (11.1)	48 (8.8)	49 (4.4)	58 (2.6)	70 (2.0)	85 (4.5)	9 (8.6)	332 (3.5)
**Physician Sex**								
Female	31 (26.5)	150 (27.6)	438 (39.2)	1,152 (50.9)	1,953 (56.9)	967 (51.4)	68 (64.8)	4,759 (50.3)
Male	86 (73.5)	394 (72.4)	680 (60.8)	1,113 (49.1)	1,479 (43.1)	914 (48.6)	37 (35.2)	4,703 (49.7)
**Primary Care Enrolment Model**								
Non-team capitation	8 (6.8)	126 (23.2)	317 (28.4)	727 (32.1)	1,190 (34.7)	532 (28.3)	11 (10.5)	2,911 (30.8)
Enhanced fee-for-service	19 (16.2)	155 (28.5)	297 (26.6)	457 (20.2)	876 (25.5)	725 (38.5)	23 (21.9)	2,552 (27.0)
Team-based capitation	12 (10.3)	60 (11.0)	286 (25.6)	814 (35.9)	1,044 (30.4)	321 (17.1)	9 (8.6)	2,546 (26.9)
Traditional fee-for-service	78 (66.7)	203 (37.3)	218 (19.5)	267 (11.8)	321 (9.4)	303 (16.1)	62 (59.0)	1,452 (15.3)
Missing	0 (0.0)	0 (0.0)	0 (0.0)	0 (0.0)	1 (0.0)	0 (0.0)	0 (0.0)	1 (0.0)
**Rurality of Practice (RIO)**								
Big cities	54 (46.2)	240 (44.1)	430 (38.5)	925 (40.8)	1,797 (52.4)	1,113 (59.2)	66 (62.9)	4,625 (48.9)
Small cities	19 (16.2)	146 (26.8)	296 (26.5)	643 (28.4)	1,018 (29.7)	558 (29.7)	25 (23.8)	2,705 (28.6)
Small towns	25 (21.4)	111 (20.4)	250 (22.4)	459 (20.3)	403 (11.7)	143 (7.6)	7 (6.7)	1,398 (14.8)
Rural	19 (16.2)	47 (8.6)	142 (12.7)	238 (10.5)	214 (6.2)	67 (3.6)	7 (6.7)	734 (7.8)
**Patient Panel Size**								
<100	57 (48.7)	61 (11.2)	81 (7.2)	122 (5.4)	116 (3.4)	89 (4.7)	35 (33.3)	561 (5.9)
100-499	30 (25.6)	103 (18.9)	106 (9.5)	169 (7.5)	239 (7.0)	200 (10.6)	32 (30.5)	879 (9.3)
500-999	9 (7.7)	85 (15.6)	201 (18.0)	490 (21.6)	802 (23.4)	443 (23.6)	20 (19.0)	2,050 (21.7)
1000-1499	15 (12.8)	104 (19.1)	321 (28.7)	654 (28.9)	1,152 (33.6)	520 (27.6)	9 (8.6)	2,775 (29.3)
1500-1999	-	79 (14.5)	212 (19.0)	469 (20.7)	675 (19.7)	341 (18.1)	-	1,785 (18.9)
≥2000	-	112 (20.6)	197 (17.6)	361 (15.9)	447 (13.0)	288 (15.3)	-	1,411 (14.9)
Missing	0 (0.0)	0 (0.0)	0 (0.0)	0 (0.0)	1 (0.0)	0 (0.0)	0 (0.0)	1 (0.0)

© 2023 Kiran T et al. *JAMA Network Open.*

eAppendix 10. Characteristics of Patients Attached to Comprehensive Family Physicians by the Percent of Physician Primary Care Provided Virtually, between February 1 and October 31, 2021, in Ontario, Canada.

	Percent Virtual Care of Primary Care Physician							
**Characteristic, n (%)**	**0%**	**>0-20%**	**>20-40%**	**>40-60%**	**>60-80%**	**>80-<100**	**100%**	**Total**
**No.**	N=46,747	N=661,761	N=1,476,716	N=2,966,294	N=4,422,486	N=2,386,349	N=51,668	N=12,012,021
**Age**								
≤18	5,900 (12.6)	115,509 (17.5)	277,180 (18.8)	567,973 (19.1)	812,766 (18.4)	394,966 (16.6)	8,678 (16.8)	2,182,972 (18.2)
19-29	6,510 (13.9)	92,402 (14.0)	200,461 (13.6)	388,821 (13.1)	595,219 (13.5)	340,662 (14.3)	8,594 (16.6)	1,632,669 (13.6)
30-44	8,980 (19.2)	131,614 (19.9)	289,588 (19.6)	585,789 (19.7)	919,525 (20.8)	520,693 (21.8)	12,330 (23.9)	2,468,519 (20.6)
45-64	13,929 (29.8)	188,939 (28.6)	407,221 (27.6)	827,379 (27.9)	1,251,073 (28.3)	696,286 (29.2)	13,555 (26.2)	3,398,382 (28.3)
65-74	6,027 (12.9)	75,225 (11.4)	167,328 (11.3)	337,959 (11.4)	480,164 (10.9)	250,916 (10.5)	4,995 (9.7)	1,322,614 (11.0)
≥75	5,401 (11.6)	58,072 (8.8)	134,938 (9.1)	258,373 (8.7)	363,739 (8.2)	182,826 (7.7)	3,516 (6.8)	1,006,865 (8.4)
**Sex**								
Female	21,331 (45.6)	321,021 (48.5)	741,288 (50.2)	1,536,715 (51.8)	2,352,148 (53.2)	1,245,292 (52.2)	27,910 (54.0)	6,245,705 (52.0)
Male	25,416 (54.4)	340,740 (51.5)	735,428 (49.8)	1,429,579 (48.2)	2,070,338 (46.8)	1,141,057 (47.8)	23,758 (46.0)	5,766,316 (48.0)
**Neighbourhood-level income quintile**								
1 (lowest)	10,563 (22.6)	158,908 (24.0)	304,381 (20.6)	531,181 (17.9)	736,255 (16.6)	447,800 (18.8)	15,183 (29.4)	2,204,271 (18.4)
2	9,165 (19.6)	141,667 (21.4)	300,837 (20.4)	567,104 (19.1)	825,310 (18.7)	470,882 (19.7)	11,060 (21.4)	2,326,025 (19.4)
3	8,990 (19.2)	135,671 (20.5)	308,776 (20.9)	611,370 (20.6)	895,940 (20.3)	487,454 (20.4)	9,859 (19.1)	2,458,060 (20.5)
4	9,589 (20.5)	121,326 (18.3)	294,905 (20.0)	631,854 (21.3)	947,742 (21.4)	490,899 (20.6)	7,797 (15.1)	2,504,112 (20.8)
5 (highest)	8,194 (17.5)	102,309 (15.5)	264,254 (17.9)	617,078 (20.8)	1,006,194 (22.8)	483,467 (20.3)	7,648 (14.8)	2,489,144 (20.7)
Missing	246 (0.5)	1,880 (0.3)	3,563 (0.2)	7,707 (0.3)	11,045 (0.2)	5,847 (0.2)	121 (0.2)	30,409 (0.3)
**Material Deprivation quintile**								
1 (least deprived)	10,154 (21.7)	110,985 (16.8)	281,815 (19.1)	687,175 (23.2)	1,184,049 (26.8)	577,501 (24.2)	10,814 (20.9)	79,839 (0.7)
2	8,765 (18.7)	123,833 (18.7)	301,672 (20.4)	646,076 (21.8)	980,348 (22.2)	511,812 (21.4)	8,416 (16.3)	2,862,493 (23.8)
3	8,331 (17.8)	124,415 (18.8)	296,005 (20.0)	568,649 (19.2)	826,842 (18.7)	453,413 (19.0)	9,163 (17.7)	2,580,922 (21.5)
4	8,651 (18.5)	134,756 (20.4)	290,618 (19.7)	528,426 (17.8)	721,805 (16.3)	421,322 (17.7)	9,757 (18.9)	2,286,818 (19.0)
5 (most deprived)	9,446 (20.2)	160,018 (24.2)	295,382 (20.0)	512,930 (17.3)	684,187 (15.5)	411,430 (17.2)	13,221 (25.6)	2,115,335 (17.6)
Missing	1,400 (3.0)	7,754 (1.2)	11,224 (0.8)	23,038 (0.8)	25,255 (0.6)	10,871 (0.5)	297 (0.6)	2,086,614 (17.4)
**Ethnic Diversity quintile**								
1 (least diverse)	7,156 (15.3)	115,340 (17.4)	292,122 (19.8)	552,978 (18.6)	558,944 (12.6)	186,743 (7.8)	4,143 (8.0)	79,839 (0.7)
2	7,169 (15.3)	116,771 (17.6)	285,005 (19.3)	576,798 (19.4)	682,543 (15.4)	256,267 (10.7)	4,099 (7.9)	1,717,426 (14.3)
3	8,341 (17.8)	109,844 (16.6)	250,549 (17.0)	559,715 (18.9)	823,205 (18.6)	385,319 (16.1)	7,750 (15.0)	1,928,652 (16.1)
4	10,814 (23.1)	126,308 (19.1)	259,401 (17.6)	578,766 (19.5)	1,017,812 (23.0)	567,822 (23.8)	12,563 (24.3)	2,144,723 (17.9)
5 (most diverse)	11,867 (25.4)	185,744 (28.1)	378,415 (25.6)	674,999 (22.8)	1,314,727 (29.7)	979,327 (41.0)	22,816 (44.2)	2,573,486 (21.4)

© 2023 Kiran T et al. *JAMA Network Open.*

Missing	1,400 (3.0)	7,754 (1.2)	11,224 (0.8)	23,038 (0.8)	25,255 (0.6)	10,871 (0.5)	297 (0.6)	3,567,895 (29.7)
**Recent Registrant (<10 years)**								
Yes	2,837 (6.1)	64,105 (9.7)	133,551 (9.0)	218,543 (7.4)	385,788 (8.7)	252,241 (10.6)	6,906 (13.4)	1,063,971 (8.9)
No	40,843 (87.4)	535,101 (80.9)	1,190,507 (80.6)	2,433,106 (82.0)	3,595,887 (81.3)	1,929,755 (80.9)	40,066 (77.5)	9,765,265 (81.3)
Missing	3,067 (6.6)	62,555 (9.5)	152,658 (10.3)	314,645 (10.6)	440,811 (10.0)	204,353 (8.6)	4,696 (9.1)	1,182,785 (9.8)
**Morbidity (Resource Utilization Band)**								
0 (non-user)	3,042 (6.5)	53,011 (8.0)	116,461 (7.9)	249,471 (8.4)	360,035 (8.1)	194,220 (8.1)	3,361 (6.5)	979,601 (8.2)
1	2,626 (5.6)	44,215 (6.7)	96,865 (6.6)	188,203 (6.3)	265,028 (6.0)	137,891 (5.8)	3,150 (6.1)	737,978 (6.1)
2	9,685 (20.7)	142,365 (21.5)	318,821 (21.6)	629,548 (21.2)	918,005 (20.8)	480,722 (20.1)	10,875 (21.0)	2,510,021 (20.9)
3	22,524 (48.2)	308,709 (46.6)	683,961 (46.3)	1,368,890 (46.1)	2,085,460 (47.2)	1,148,901 (48.1)	25,066 (48.5)	5,643,511 (47.0)
4	5,898 (12.6)	80,837 (12.2)	186,546 (12.6)	380,537 (12.8)	579,236 (13.1)	312,847 (13.1)	6,868 (13.3)	1,552,769 (12.9)
5 (highest user)	2,972 (6.4)	32,624 (4.9)	74,062 (5.0)	149,645 (5.0)	214,722 (4.9)	111,768 (4.7)	2,348 (4.5)	588,141 (4.9)
**Co-Morbidity (Adjusted Diagnoses Groups)**								
0	3,058 (6.5)	53,201 (8.0)	116,978 (7.9)	250,775 (8.5)	361,757 (8.2)	194,831 (8.2)	3,366 (6.5)	983,966 (8.2)
1-4	24,958 (53.4)	337,144 (50.9)	753,137 (51.0)	1,488,427 (50.2)	2,161,033 (48.9)	1,136,125 (47.6)	25,142 (48.7)	5,925,966 (49.3)
5-9	14,917 (31.9)	220,459 (33.3)	493,326 (33.4)	999,668 (33.7)	1,540,214 (34.8)	849,104 (35.6)	18,600 (36.0)	4,136,288 (34.4)
≥ 10	3,814 (8.2)	50,957 (7.7)	113,275 (7.7)	227,424 (7.7)	359,482 (8.1)	206,289 (8.6)	4,560 (8.8)	965,801 (8.0)
**Chronic Conditions**								
Hypertension	13,146 (28.1)	161,768 (24.4)	350,579 (23.7)	676,513 (22.8)	972,746 (22.0)	525,617 (22.0)	10,873 (21.0)	2,711,242 (22.6)
DM	6,821 (14.6)	82,520 (12.5)	175,651 (11.9)	330,527 (11.1)	478,984 (10.8)	273,799 (11.5)	5,977 (11.6)	1,354,279 (11.3)
CHF	1,978 (4.2)	13,945 (2.1)	31,544 (2.1)	59,835 (2.0)	79,818 (1.8)	39,321 (1.6)	820 (1.6)	227,261 (1.9)
AMI	714 (1.5)	8,019 (1.2)	17,897 (1.2)	34,266 (1.2)	44,602 (1.0)	21,978 (0.9)	401 (0.8)	127,877 (1.1)
Asthma	7,405 (15.8)	102,415 (15.5)	226,508 (15.3)	452,736 (15.3)	690,691 (15.6)	375,572 (15.7)	8,202 (15.9)	1,863,529 (15.5)
COPD	3,865 (8.3)	48,970 (7.4)	111,406 (7.5)	210,552 (7.1)	266,783 (6.0)	131,864 (5.5)	2,725 (5.3)	776,165 (6.5)
Mental health	11,249 (24.1)	131,914 (19.9)	302,105 (20.5)	611,709 (20.6)	946,647 (21.4)	515,357 (21.6)	12,559 (24.3)	2,531,540 (21.1)
**Rurality**								
Big cities	20,502 (43.9)	261,133 (39.5)	513,917 (34.8)	1,065,398 (35.9)	1,951,424 (44.1)	1,196,905 (50.2)	32,308 (62.5)	5,041,587 (42.0)
Small cities	13,519 (28.9)	201,308 (30.4)	421,487 (28.5)	907,415 (30.6)	1,485,352 (33.6)	833,369 (34.9)	12,408 (24.0)	3,874,858 (32.3)
Small towns	7,545 (16.1)	148,806 (22.5)	389,245 (26.4)	727,505 (24.5)	746,817 (16.9)	279,701 (11.7)	3,510 (6.8)	2,303,129 (19.2)
Rural	5,181 (11.1)	50,514 (7.6)	152,067 (10.3)	265,976 (9.0)	238,893 (5.4)	76,374 (3.2)	3,442 (6.7)	792,447 (6.6)

© 2023 Kiran T et al. *JAMA Network Open.*

eAppendix 11. Health Service Utilization of Patients Attached to Comprehensive Family Physicians Stratified by the Attached Physician's Percent of Care Provided Virtually, between February 1 and October 2021, in Ontario Canada.

**Physician % Virtual Care**	**0%**	**>0-20%**	**>20-40%**	**>40-60%**	**>60-80%**	**>80-<100%**	**100%**	**Total**
**No. Patients**	N=46,747	N=661,761	N=1,476,716	N=2,966,294	N=4,422,486	N=2,386,349	N=51,668	N=12,125,959
**Primary Care Services**								
Any primary care visit, n (%)	26,862 (57.5)	411,991 (62.3)	921,775 (62.4)	1,858,118 (62.6)	2,855,323 (64.6)	1,569,605 (65.8)	32,277 (62.5)	7,675,951 (63.9)
Number of primary care visits per patient, mean ± SD	2.3 ± 4.0	2.4 ± 3.9	2.4 ± 3.8	2.3 ± 3.6	2.6 ± 3.8	2.9 ± 4.2	2.8 ± 4.5	2.5 ± 3.9
Percent of visits with usual family physician[a], mean ± SD	53.3 ± 45.2	67.7 ± 39.8	70.3 ± 38.1	71.7 ± 36.8	71.8 ± 36.6	68.2 ± 38.8	40.0 ± 43.0	70.4 ± 37.6
**Other Health Care Use**								
Any specialist visits, n (%)	18,601 (39.8)	249,150 (37.6)	563,324 (38.1)	1,150,948 (38.8)	1,763,140 (39.9)	959,637 (40.2)	20,368 (39.4)	4,725,168 (39.3)
Ambulatory care-sensitive condition visits, n (%)	164 (0.4)	1,734 (0.3)	3,675 (0.2)	6,968 (0.2)	9,030 (0.2)	4,393 (0.2)	91 (0.2)	26,055 (0.2)
**Emergency Department Utilization, February to October 2019/2021**								
Total visits per 1,000, 2021, mean ± SD[b]	348.0 ± 1204.0	287.2 ± 981.7	282.4 ± 1005.9	274.7 ± 893.5	248.9 ± 810.0	238.0 ± 780.5	262.1 ± 972.0	259.8 ± 864.4
Total visits per 1,000, 2019, mean ± SD[b]	387.9 ± 1293.1	335.5 ± 1023.4	330.3 ± 959.2	317.4 ± 945.7	284.1 ± 911.7	268.7 ± 828.6	282.2 ± 853.4	298.4 ± 920.0
Absolute difference 2021 vs 2019 (95% CI)	-39.9 (-54.3, -25.6)	-48.3 (-51.8, -44.9)	-47.9 (-50.2, -45.6)	-42.7 (-44.2, -41.2)	-35.2 (-36.4, -34.0)	-30.8 (-32.3, -29.3)	-20.0 (-30.2, -9.9)	-38.6 (-39.3, -37.9)
Percent change 2021 vs 2019 (95% CI)	-10.3 (-13.8, -6.8)	-14.4 (-15.4, -13.4)	-14.5 (-15.2, -13.9)	-13.5 (-13.9, -13.0)	-12.4 (-12.8, -12.0)	-11.5 (-12.0, -10.9)	-7.1 (-10.7, -3.5)	-12.9 (-13.2, -12.7)

[a] Among patients with 2 or more visits between February and October 2021

[b] This includes only patients whose attached physicians had claims in both 2021 and 2019.

Abbreviations: SD, standard deviation; CI, confidence interval

© 2023 Kiran T et al. *JAMA Network Open.*
