# Supplementary material for: Virtual Care and Emergency Department Use During the COVID-19 Pandemic Among Patients of Family Physicians in Ontario, Canada
Source: JAMA Netw Open. 2023 Apr 28;6(4):e239602. doi: 10.1001/jamanetworkopen.2023.9602 (PMC10148195; doi:10.1001/jamanetworkopen.2023.9602)
Supplement: Supplement 2. — Data Sharing Statement [file jamanetwopen-e239602-s002.pdf]

Data Sharing Statement

Kiran. Virtual Care and Emergency Department Use During the COVID-19 Pandemic Among Patients of Family Physicians in Ontario, Canada. *JAMA Netw Open*. Published April 28, 2023. doi:10.1001/jamanetworkopen.2023.9602

Data
Data available: No

Additional Information
Explanation for why data not available: The data sets from this study are held securely in coded form at ICES. Data-sharing agreements prohibit ICES from making the data sets publicly available, but access may be granted to those who meet pre-specified criteria for confidential access, available at www.ices.on.ca/DAS. The complete data set creation plan, and underlying analytic code are available from the authors upon request, understanding that the programs may rely upon coding templates or macros unique to ICES.
